# Supplementary material for: Mental disorders, COVID-19-related life-saving measures and mortality in France: A nationwide cohort study
Source: PLoS Med. 2023 Feb 6;20(2):e1004134. doi: 10.1371/journal.pmed.1004134 (PMC10089350; doi:10.1371/journal.pmed.1004134)
Supplement: S1 Text — Appendix A. Coding dictionary. Appendix B. RECORD statement1 –checklist of items, extended from the STROBE statement, for observational studies using routinely-collected health data. Table A. Characteristics of inpatients with symptomatic COVID-19 by pandemic period (n = 465,750). Table B. 120-day mortality risk of inpatients with symptomatic COVID-19 by salvage therapy triage, univariate analyses (n = 465,750). Table C. 120-day mortality risk of inpatients with symptomatic COVID-19 by salvage therapy triage, simultaneous probit multivariate model (n = 465,750). Table D. Controlled direct effects of preexisting mental disorders on 120-day mortality risk of inpatients with symptomatic COVID-19, causal mediation analyses (n = 465,750). Table E. Characteristics of inpatients with symptomatic COVID-19 by preexisting mental disorders (n = 465,750). Table F. Characteristics of inpatients with symptomatic COVID-19 by age category and preexisting mental disorders (n = 465,750). Table G. 120-day mortality and salvage therapy risks by category of preexisting mental disorders (n = 465,750). Fig A. Associations of pandemic periods and preexisting mental disorders with 120-day mortality risk among inpatients with symptomatic COVID-19 aged 18–64 years (n = 164,591). Fig B. Associations of pandemic periods and preexisting mental disorders with 120-day mortality risk among inpatients with symptomatic COVID-19 aged 65 years and above (n = 301,159). Fig C. Associations of pandemic periods and preexisting mental disorders with salvage therapy rate among inpatients with symptomatic COVID-19 aged 18–64 years (n = 164,591). Fig D. Associations of pandemic periods and preexisting mental disorders with salvage therapy rate among inpatients with symptomatic COVID-19 aged 65 years and above (n = 301,159). Fig E. Associations of pandemic periods and preexisting mental disorders with 120-day mortality risk among inpatients with COVID-19-related respiratory symptoms (n = 371,016). Fig F. Associ [file pmed.1004134.s001.docx]

**Mental disorders, COVID-19-related life-saving measures and mortality in France: a nationwide cohort study.**

Schwarzinger et al., PLoS medicine 2022

Contents

[Appendix A. Coding dictionary 3](#_Toc126570280)

[Appendix B. RECORD statement^1^ – checklist of items, extended from the STROBE statement, for observational studies using routinely-collected health data 8](#_Toc126570281)

[Table A. Characteristics of inpatients with symptomatic COVID-19 by pandemic period (n=465 750) 13](#_Toc126570282)

[Table B. 120-day mortality risk of inpatients with symptomatic COVID-19 by salvage therapy triage, univariate analyses (n=465 750) 16](#_Toc126570283)

[Table C. 120-day mortality risk of inpatients with symptomatic COVID-19 by salvage therapy triage, simultaneous probit multivariate model (n=465 750) 19](#_Toc126570284)

[Table D. Controlled direct effects of pre-existing mental disorders on 120-day mortality risk of inpatients with symptomatic COVID-19, causal mediation analyses (n=465 750) 22](#_Toc126570285)

[Table E. Characteristics of inpatients with symptomatic COVID-19 by pre-existing mental disorders (n=465 750) 23](#_Toc126570286)

[Table F. Characteristics of inpatients with symptomatic COVID-19 by age category and pre-existing mental disorders (n=465 750) 26](#_Toc126570287)

[Table G. 120-day mortality and salvage therapy risks by category of pre-existing mental disorders (n=465 750) 29](#_Toc126570288)

[Fig A. Associations of pandemic periods and pre-existing mental disorders with 120-day mortality risk among inpatients with symptomatic COVID-19 aged 18-64 years (n=164 591) 30](#_Toc126570289)

[Fig B. Associations of pandemic periods and pre-existing mental disorders with 120-day mortality risk among inpatients with symptomatic COVID-19 aged 65 years and above (n=301 159) 31](#_Toc126570290)

[Fig C. Associations of pandemic periods and pre-existing mental disorders with salvage therapy rate among inpatients with symptomatic COVID-19 aged 18-64 years (n=164 591) 32](#_Toc126570291)

[Fig D. Associations of pandemic periods and pre-existing mental disorders with salvage therapy rate among inpatients with symptomatic COVID-19 aged 65 years and above (n=301 159) 33](#_Toc126570292)

[Fig E. Associations of pandemic periods and pre-existing mental disorders with 120-day mortality risk among inpatients with COVID-19-related respiratory symptoms (n=371 016) 34](#_Toc126570293)

[Fig F. Associations of pandemic periods and pre-existing mental disorders on salvage therapy rate among inpatients with COVID-19-related respiratory symptoms (n=371 016) 35](#_Toc126570294)

[Fig G. Associations of pandemic periods and pre-existing mental disorders with 120-day mortality risk among inpatients admitted for symptomatic COVID-19 (n= 395 323) 36](#_Toc126570295)

[Fig H. Associations of pandemic periods and pre-existing mental disorders on salvage therapy rate among inpatients admitted for symptomatic COVID-19 (n= 395 323) 37](#_Toc126570296)

[Fig I. Associations of pandemic periods and pre-existing mental disorders with intensive-care unit admission rate among inpatients with symptomatic COVID-19 (n=465 750) 38](#_Toc126570297)

[Fig J. Associations of pandemic periods and pre-existing mental disorders with 28-day mortality risk among inpatients with symptomatic COVID-19 (n=465 750) 39](#_Toc126570298)

[Fig K. Associations of pandemic periods and pre-existing mental disorders with mortality risk at first acute hospital discharge among inpatients with symptomatic COVID-19 (n=465 750) 40](#_Toc126570299)

#

# Appendix A. Coding dictionary

|  | | **ICD-10-FR[1] or French Diagnosis-related group (DRG)** | **Reference** |
| --- | --- | --- | --- |
| **COVID-19 with symptoms** | | U07.10, U07.11, U07.12, U07.14, U07.15, U10.9 | [2] |
|  | COVID-19 with respiratory symptoms | U07.10, U07.11 |  |
|  | COVID-19-related multisystem inflammatory syndrome | COVID-19 and R65 in the follow-up; or U10.9 (starting October 2020) |  |
| **Sociodemographic factors** | | Assessed at hospital entry of first hospital stay with COVID-19 record |  |
|  | Fdep deprivation index | FDep deprivation index is based on the median household income, the percentage high school graduates in the population aged 15 years and older, the percentage bluecollar workers in the active population, and the unemployment rate. FDep is available for 2015 at the commune level (n=35 322) and the weighted mean was computed at the postal code level (n=5783) with use of 2015 French census. | [3-5] |
|  | Main region of residency | Based on region of residency |  |
| **Mental disorders** | |  |  |
|  | Dementia | F00x, F01x, F02x, F03x, G30x, F05.1, G31.1 | [6-8] |
|  | Depression | As primary disorder: F32x, F33x,  As secondary disorder: F31.3, F31.4, F31.5, F31.6,  F06.32, F20.4, F25.1, F41.2, F53.0, F55+0, F92.0,  F10.54, F11.54, F12.54, F13.54, F14.54, F15.54, F16.54, F17.54, F18.54, F19.54 | [9] |
|  | Anxiety disorders | F4xx | ICD-10 label |
|  | Schizophrenia | As primary disorder: F2xx,  As secondary disorder: F10.5, F11.5, F12.5, F13.5, F14.5, F15.5, F16.5, F17.5, F18.5, F19.5 | [9] |
|  | Alcohol use disorders | F10x, Z502,  E24.4, I42.6, K70x, K29.2, K85.2, K86.0, G31.2, G62.1, G72.1, O35.4 R780, T51, X45, X65, Y15, Y90, Y91, Z714, Z721 | [9, 10] |
|  | Opioid addiction | F11x, T40.0, T40.1, T40.2, T40.3, T40.4 | ICD-10 label |
|  | Down syndrome | Q90 | [9] |
|  | Other learning disabilities | F7xx NOT Q90 | [9] |
|  | Other disorder with psychiatric ward admission | admission in acute hospital with DRG 19xxxx (psychiatric care) or psychiatric hospital NOT previous mental disorders |  |
| **Risk factors for severe COVID-19** | |  |  |
|  | Tobacco smoking | F17x, T65.2, Z71.6, Z72.0 | [9] |
|  | Obesity (BMI≥30 kg/m^2^) | E66x NOT E66.03, E66.13, E66.83, E66.93 | [9] |
|  | Hypertension | I10x, I11x, I12x, I13x, I674, O10x, O11x, O13x, O14x, O15x, O16x | [9] |
|  | Diabetes mellitus | E10x, E11x, E12x, E13x, E14x,  G59.0, G63.2, H28.0, H36.0, I79.2, M14.2, N08.3, O24x | [9] |
| **Severe physical comorbidities** | |  |  |
|  | Congestive heart failure | I09.9, I11.0, I13.0, I13.2, I25.5, I42.0, I42.5, I42.6, I42.7, I42.8, I42.9,  I43x, I50x, P29.0 | [6-8] |
|  | Peripheral vascular disease | I70x, I71x, I73.1, I73.8, I73.9, I77.1, I79.0, I79.2,  K55.1, K55.8, K55.9, Z95.8, Z95.9 | [6-8] |
|  | Cerebrovascular disease | I6xx G45x, G46x, H34.0 | [6-8] |
|  | Chronic pulmonary disease (including asthma) | J4xx, J60x, J61x, J62x, J63x, J64x, J65x, J66x, J67x, J68.4,  J70.1, J70.3, I27.8, I27.9 | [6-8] |
|  | Hemiplegia | G04.1, G11.4, G80.1, G80.2, G81x, G82x,  G83.0, G83.1, G83.2, G83.3, G83.4, G83.9 | [6-8] |
|  | Moderate or severe liver disease | I85.0, I85.9, I86.4, I98.2, K70.4, K71.1, K72.1, K72.9, K76.5, K76.6, K76.7 | [6-8] |
|  | Mild liver disease | B18x, K70.0, K70.1, K70.2, K70.3, K70.9, K71.3, K71.4, K71.5, K71.7,  K73x, K74x, K76.0, K76.2, K76.3, K76.4, K76.8, K76.9, Z94.4 | [6-8] |
|  | Moderate or severe renal disease | I12.0, I13.1, N03.2, N03.3, N03.4, N03.5, N03.6, N03.7, N05.2, N05.3, N05.4, N05.5, N05.6, N05.7,  N18.0, N18.3, N18.4, N18.5, N18.8, N18.9, N19x, N25.0,  Z49x, Z94.0, Z99.2  OR admission in acute hospital with DRG 28Z01x, 28Z02x, 28Z03x, 28Z04x, 11K02x (dialysis) | [6-8] |
|  | Metastatic solid tumour | C77x, C78x, C79x, C80x | [6-8] |
|  | Solid tumour without metastasis | Cxx, B21.0, B21.7, B21.8, B21.9) NOT C44x, C77x, C78x, C79x, C80x,  C81x, C82x, C83x, C84x, C85x, C86x, C87x, C88x, C89x, C90x, C91x, C92x, C93x, C94x, C95x, C96x | [6-8] |
|  | Haematological malignancy (disentangled from solid tumor) | C81x, C82x, C83x, C84x, C85x, C86x, C87x, C88x, C89x, C90x, C91x, C92x, C93x, C94x, C95x, C96x | [6-8] |
|  | AIDS | B20x, B21x, B22x, B24x | [6-8] |
|  | Transplant recipient | Z94x, T86x OR admission in acute hospital with DRG 27xxxx (transplantation) |  |
| **Salvage therapy** | |  |  |
|  | Intensive care unit | Medical unit code: 01, 06, 13 |  |
|  | Extracorporeal membrane oxygenation (ECMO) | GLJF010 | French medical procedure (CCAM) classification |
|  | Invasive mechanical respiratory support | GLLD004, GLLD005, GLLD006, GLLD007, GLLD008, GLLD009, GLLD012,  GLLD013, GLLD015, DKMD001, GEPA004, GELD002, GELD004 |  |
|  | Continuous positive airway pressure (CPAP) | GLLD003 |  |

Reference

1. Agence Technique de l'Information Hospitalière. Les mises à jour de la CIM-10 depuis 2006 [Updates of ICD-10-FR since 2006] (Available from: <https://www.atih.sante.fr/nomenclatures-de-recueil-de-linformation/cim>; Accessed: 04/01/2022). 2022.

2. Agence Technique de l'Information Hospitalière. Mise à jour des consignes de codage des séjours COVID-19 [Coding instructions and updates for COVID-19 hospital stays] (Available from: <https://www.atih.sante.fr/mise-jour-des-consignes-de-codage-des-sejours-covid-19>; Accessed: 04/01/2022). 2021.

3. Rey G, Jougla E, Fouillet A, Hemon D. Ecological association between a deprivation index and mortality in France over the period 1997 - 2001: variations with spatial scale, degree of urbanicity, age, gender and cause of death. BMC public health. 2009;9:33.

4. Ghosn W, Kassie D, Jougla E, Salem G, Rey G, Rican S. Trends in geographic mortality inequalities and their association with population changes in France, 1975-2006. European journal of public health. 2013;23(5):834-40.

5. Inserm CépiDc. Indicateurs écologiques du niveau socio-économique [Ecological indicators of the socio-economic level] (Available from: <https://www.cepidc.inserm.fr/documentation/indicateurs-ecologiques-du-niveau-socio-economique>; Accessed: 04/01/2022). 2015.

6. Charlson ME, Pompei P, Ales KL, MacKenzie CR. A new method of classifying prognostic comorbidity in longitudinal studies: development and validation. Journal of chronic diseases. 1987;40(5):373-83.

7. Quan H, Sundararajan V, Halfon P, Fong A, Burnand B, Luthi JC, et al. Coding algorithms for defining comorbidities in ICD-9-CM and ICD-10 administrative data. Medical care. 2005;43(11):1130-9.

8. Bannay A, Chaignot C, Blotiere PO, Basson M, Weill A, Ricordeau P, et al. The Best Use of the Charlson Comorbidity Index With Electronic Health Care Database to Predict Mortality. Medical care. 2016;54(2):188-94.

9. Schwarzinger M, Pollock BG, Hasan OSM, Dufouil C, Rehm J, QalyDays Study G. Contribution of alcohol use disorders to the burden of dementia in France 2008-13: a nationwide retrospective cohort study. The Lancet Public health. 2018;3(3):e124-e32.

10. Rehm J, Mathers C, Popova S, Thavorncharoensap M, Teerawattananon Y, Patra J. Global burden of disease and injury and economic cost attributable to alcohol use and alcohol-use disorders. Lancet. 2009;373(9682):2223-33.

# Appendix B. RECORD statement^1^ – checklist of items, extended from the STROBE statement, for observational studies using routinely-collected health data

|  | **Item No.** | **STROBE items** | **Location in manuscript where items are reported** | **RECORD items** | **Location in manuscript where items are reported** |
| --- | --- | --- | --- | --- | --- |
| **Title and abstract** | | | | | |
|  | 1 | (a) Indicate the study’s design with a commonly used term in the title or the abstract (b) Provide in the abstract an informative and balanced summary of what was done and what was found | a) Done [Title]  b) Done [Abstract] | RECORD 1.1: The type of data used should be specified in the title or abstract. When possible, the name of the databases used should be included.  RECORD 1.2: If applicable, the geographic region and timeframe within which the study took place should be reported in the title or abstract.  RECORD 1.3: If linkage between databases was conducted for the study, this should be clearly stated in the title or abstract. | 1.1) Done [Abstract/Methods] The full name of the database is provided in Methods.  1.2) Done [Title/Abstract]  1.3) Not applicable. |
| **Introduction** | | | | | |
| Background rationale | 2 | Explain the scientific background and rationale for the investigation being reported | Done [Introduction] | | |
| Objectives | 3 | State specific objectives, including any prespecified hypotheses | Done [Introduction] | | |
| **Methods** | | | | | |
| Study Design | 4 | Present key elements of study design early in the paper | Done [Title; Abstract; Methods] | | |
| Setting | 5 | Describe the setting, locations, and relevant dates, including periods of recruitment, exposure, follow-up, and data collection | Done [Title; Abstract; Methods] | | |
| Participants | 6 | *(a) Cohort study* - Give the eligibility criteria, and the sources and methods of selection of participants. Describe methods of follow-up  *Case-control study* - Give the eligibility criteria, and the sources and methods of case ascertainment and control selection. Give the rationale for the choice of cases and controls  *Cross-sectional study* - Give the eligibility criteria, and the sources and methods of selection of participants  *(b) Cohort study* - For matched studies, give matching criteria and number of exposed and unexposed  *Case-control study* - For matched studies, give matching criteria and the number of controls per case | a) Done [Methods]  b) Not applicable. | RECORD 6.1: The methods of study population selection (such as codes or algorithms used to identify subjects) should be listed in detail. If this is not possible, an explanation should be provided.  RECORD 6.2: Any validation studies of the codes or algorithms used to select the population should be referenced. If validation was conducted for this study and not published elsewhere, detailed methods and results should be provided.  RECORD 6.3: If the study involved linkage of databases, consider use of a flow diagram or other graphical display to demonstrate the data linkage process, including the number of individuals with linked data at each stage. | 6.1) Done [Methods]. Details of ICD-10 codes used are provided in S1 Appendix.  6.2) Reference provided in S1 Appendix.  The robustness of the main study results were studied in sensitivity analyses with different selection criteria [Methods; Results].  6.3) Not applicable |
| Variables | 7 | Clearly define all outcomes, exposures, predictors, potential confounders, and effect modifiers. Give diagnostic criteria, if applicable. | Done [Methods]. | RECORD 7.1: A complete list of codes and algorithms used to classify exposures, outcomes, confounders, and effect modifiers should be provided. If these cannot be reported, an explanation should be provided. | A complete list of codes and references are provided in S1Appendix. |
| Data sources/ measurement | 8 | For each variable of interest, give sources of data and details of methods of assessment (measurement).  Describe comparability of assessment methods if there is more than one group | All variables were identified from the same source of data [Methods; S1Appendix].  Not applicable. | | |
| Bias | 9 | Describe any efforts to address potential sources of bias | The robustness of the main study results were studied in sensitivity analyses with different outcome definitions [Methods; Results] | | |
| Study size | 10 | Explain how the study size was arrived at | Not applicable: the study included all patients recorded with COVID-19 identified in the French National Hospital Discharge database [Methods]. | | |
| Quantitative variables | 11 | Explain how quantitative variables were handled in the analyses. If applicable, describe which groupings were chosen, and why | Not applicable (only binary or categorical variables). | | |
| Statistical methods | 12 | (a) Describe all statistical methods, including those used to control for confounding  (b) Describe any methods used to examine subgroups and interactions  (c) Explain how missing data were addressed  (d) *Cohort study* - If applicable, explain how loss to follow-up was addressed  *Case-control study* - If applicable, explain how matching of cases and controls was addressed  *Cross-sectional study* - If applicable, describe analytical methods taking account of sampling strategy  (e) Describe any sensitivity analyses | a) Done [Methods]  b) The robustness of the main study results were studied for two age groups and each category of mental disorder [Methods; Results].  c) Not applicable. Coding bias was discussed in the limitations [Discussion].  d) Not applicable as all patients were followed with use of all hospital discharges over a fixed period of time [Methods].  e) Multiple sensitivity analyses were conducted on patient selection and outcome definition [Methods; Results]. All results of sensitivity analyses are provided in Appendix Tables and Figures. | | |
| Data access and cleaning methods |  | .. |  | RECORD 12.1: Authors should describe the extent to which the investigators had access to the database population used to create the study population.  RECORD 12.2: Authors should provide information on the data cleaning methods used in the study. | 12.1) Done [Methods]  12.2) No data cleaning methods were used to define exposures (i.e., a sensitive rather than specific approach). |
| Linkage |  | .. |  | RECORD 12.3: State whether the study included person-level, institutional-level, or other data linkage across two or more databases. The methods of linkage and methods of linkage quality evaluation should be provided. | The study additionally included linkage on postal codes of residency to control for area deprivation index [Methods; S1 Appendix]. |
| **Results** | | | | | |
| Participants | 13 | (a) Report the numbers of individuals at each stage of the study (*e.g.*, numbers potentially eligible, examined for eligibility, confirmed eligible, included in the study, completing follow-up, and analysed)  (b) Give reasons for non-participation at each stage.  (c) Consider use of a flow diagram | a) Done [Results]  b) Not applicable | RECORD 13.1: Describe in detail the selection of the persons included in the study (*i.e.,* study population selection) including filtering based on data quality, data availability and linkage. The selection of included persons can be described in the text and/or by means of the study flow diagram. | Done [Methods; Results]. The robustness of the main study results were studied in sensitivity analyses with different selection criteria [Methods; Results]. |
| Descriptive data | 14 | (a) Give characteristics of study participants (*e.g.*, demographic, clinical, social) and information on exposures and potential confounders  (b) Indicate the number of participants with missing data for each variable of interest  (c) *Cohort study* - summarise follow-up time (*e.g.*, average and total amount) | a) Done [Tables 1; S1 Table by period]  b) Not applicable  c) Not applicable: all patients were followed after first COVID-19 diagnosis record at hospital with use of all hospital discharges over 120 days [Methods]. | | |
| Outcome data | 15 | *Cohort study* - Report numbers of outcome events or summary measures over time  *Case-control study* - Report numbers in each exposure category, or summary measures of exposure  *Cross-sectional study* - Report numbers of outcome events or summary measures | Done [Tables 1 and 2 for mortality and salvage therapy, respectively; S1 Table by period]. | | |
| Main results | 16 | (a) Give unadjusted estimates and, if applicable, confounder-adjusted estimates and their precision (e.g., 95% confidence interval). Make clear which confounders were adjusted for and why they were included  (b) Report category boundaries when continuous variables were categorized  (c) If relevant, consider translating estimates of relative risk into absolute risk for a meaningful time period | a) Done [Tables 1 and 2 for mortality and salvage therapy, respectively]. No variable selection was performed [Methods] and odd-ratios were adjusted on all variables.  b) Not applicable.  c) Done with excess risks presented in Figures 2 and 3 (and all Appendix Figures for sensitivity analyses). | | |
| Other analyses | 17 | Report other analyses done—e.g., analyses of subgroups and interactions, and sensitivity analyses | Subgroup analyses (age group; each category of mental disorder) and sensitivity analyses (patient selection; outcome definition) were conducted. All results are provided in Appendix Figures. | | |
| **Discussion** | | | | | |
| Key results | 18 | Summarise key results with reference to study objectives | Done [Discussion, first paragraph]. | | |
| Limitations | 19 | Discuss limitations of the study, taking into account sources of potential bias or imprecision. Discuss both direction and magnitude of any potential bias | Done [Discussion, 2 paragraphs] | RECORD 19.1: Discuss the implications of using data that were not created or collected to answer the specific research question(s). Include discussion of misclassification bias, unmeasured confounding, missing data, and changing eligibility over time, as they pertain to the study being reported. | Done [Discussion, 1 paragraph] |
| Interpretation | 20 | Give a cautious overall interpretation of results considering objectives, limitations, multiplicity of analyses, results from similar studies, and other relevant evidence | Done [Discussion] | | |
| Generalisability | 21 | Discuss the generalisability (external validity) of the study results | Done [Discussion] | | |
| **Other Information** | | | | | |
| Funding | 22 | Give the source of funding and the role of the funders for the present study and, if applicable, for the original study on which the present article is based | This study received no external funding [Abstract; Methods]. | | |
| Accessibility of protocol, raw data, and programming code |  |  |  | RECORD 22.1: Authors should provide information on how to access any supplemental information such as the study protocol, raw data, or programming code. | Raw data of the French National Hospital Discharge database cannot be shared without due permission from health authorities. |

Reference

1. Benchimol EI, Smeeth L, Guttmann A, Harron K, Moher D, Petersen I, Sørensen HT, von Elm E, Langan SM, the RECORD Working Committee. The REporting of studies Conducted using Observational Routinely-collected health Data (RECORD) Statement. *PLoS Medicine* 2015;12(10):e1001885.

#

# Table A. Characteristics of inpatients with symptomatic COVID-19 by pandemic period (n=465 750)

| **Risk factors and outcomes** | | **All patients** | **First wave 2020 weeks 11-19** | **First inter-wave 2020 weeks 20-36** | **Second wave 2020 week 37-2021 week 8** | **Third wave 2021 weeks 9-19** | **Second inter-wave 2021 weeks 20-30** | **Fourth wave 2021 weeks 31-34** |
| --- | --- | --- | --- | --- | --- | --- | --- | --- |
|  |  | **465 750 (100.0)** | **99 633 (21.4)** | **31 840 (6.8)** | **202 771 (43.5)** | **99 430 (21.3)** | **17 352 (3.7)** | **14 724 (3.2)** |
| COVID-19 with respiratory symptoms | | 371 016 (79.7) | 81 940 (82.2) | 11 865 (37.3) | 162 855 (80.3) | 88 084 (88.6) | 13 434 (77.4) | 12 838 (87.2) |
| COVID-19-related multisystem inflammatory syndrome | | 110 785 (23.8) | 26 660 (26.8) | 20 084 (63.1) | 45 530 (22.5) | 13 635 (13.7) | 3 442 (19.8) | 1 434 (9.7) |
| Male | | 251 360 (54.0) | 54 597 (54.8) | 17 134 (53.8) | 108 991 (53.8) | 53 571 (53.9) | 9 242 (53.3) | 7 825 (53.1) |
| Age, median (IQR) years | | 72 (58-84) | 72 (58-84) | 73 (58-84) | 75 (62-85) | 68 (55-80) | 62 (47-76) | 62 (47-77) |
|  | ≥90 years | 50 512 (10.8) | 10 500 (10.5) | 3 666 (11.5) | 26 427 (13.0) | 7 745 (7.8) | 1 178 (6.8) | 996 (6.8) |
|  | 85-89 years | 56 848 (12.2) | 11 997 (12.0) | 4 078 (12.8) | 29 181 (14.4) | 9 260 (9.3) | 1 251 (7.2) | 1 081 (7.3) |
|  | 80-84 years | 52 601 (11.3) | 11 465 (11.5) | 3 721 (11.7) | 26 123 (12.9) | 9 063 (9.1) | 1 185 (6.8) | 1 044 (7.1) |
|  | 75-79 years | 45 120 (9.7) | 9 685 (9.7) | 3 172 (10.0) | 21 716 (10.7) | 8 239 (8.3) | 1 207 (7.0) | 1 101 (7.5) |
|  | 70-74 years | 52 002 (11.2) | 10 695 (10.7) | 3 411 (10.7) | 23 346 (11.5) | 11 718 (11.8) | 1 519 (8.8) | 1 313 (8.9) |
|  | 65-69 years | 44 076 (9.5) | 9 314 (9.3) | 2 875 (9.0) | 18 336 (9.0) | 10 787 (10.8) | 1 473 (8.5) | 1 291 (8.8) |
|  | 60-64 years | 37 901 (8.1) | 8 470 (8.5) | 2 272 (7.1) | 14 948 (7.4) | 9 436 (9.5) | 1 494 (8.6) | 1 281 (8.7) |
|  | 55-59 years | 33 255 (7.1) | 7 235 (7.3) | 1 888 (5.9) | 12 298 (6.1) | 8 996 (9.0) | 1 547 (8.9) | 1 291 (8.8) |
|  | 50-54 years | 26 336 (5.7) | 5 895 (5.9) | 1 523 (4.8) | 9 147 (4.5) | 7 167 (7.2) | 1 463 (8.4) | 1 141 (7.7) |
|  | 45-49 years | 19 896 (4.3) | 4 356 (4.4) | 1 191 (3.7) | 6 571 (3.2) | 5 541 (5.6) | 1 270 (7.3) | 967 (6.6) |
|  | 40-44 years | 13 792 (3.0) | 2 977 (3.0) | 835 (2.6) | 4 272 (2.1) | 3 874 (3.9) | 975 (5.6) | 859 (5.8) |
|  | 35-39 years | 11 438 (2.5) | 2 543 (2.6) | 870 (2.7) | 3 562 (1.8) | 2 844 (2.9) | 843 (4.9) | 776 (5.3) |
|  | 30-34 years | 9 508 (2.0) | 1 987 (2.0) | 859 (2.7) | 2 896 (1.4) | 2 220 (2.2) | 820 (4.7) | 726 (4.9) |
|  | 18-29 years | 12 465 (2.7) | 2 514 (2.5) | 1 479 (4.6) | 3 948 (1.9) | 2 540 (2.6) | 1 127 (6.5) | 857 (5.8) |
| Area deprivation index quintile | |  |  |  |  |  |  |  |
|  | FDeP 5 (most deprived) | 114 480 (24.6) | 23 369 (23.5) | 7 837 (24.6) | 49 182 (24.3) | 26 352 (26.5) | 4 476 (25.8) | 3 264 (22.2) |
|  | FDeP 4 | 92 877 (19.9) | 17 885 (18.0) | 6 610 (20.8) | 42 357 (20.9) | 19 749 (19.9) | 3 416 (19.7) | 2 860 (19.4) |
|  | FDeP 3 | 85 390 (18.3) | 16 739 (16.8) | 6 020 (18.9) | 38 512 (19.0) | 17 730 (17.8) | 3 228 (18.6) | 3 161 (21.5) |
|  | FDep 2 | 84 253 (18.1) | 18 396 (18.5) | 5 804 (18.2) | 36 330 (17.9) | 17 569 (17.7) | 3 189 (18.4) | 2 965 (20.1) |
|  | FDep 1 (least deprived) | 88 750 (19.1) | 23 244 (23.3) | 5 569 (17.5) | 36 390 (17.9) | 18 030 (18.1) | 3 043 (17.5) | 2 474 (16.8) |
| Residency in metropolitan France | |  |  |  |  |  |  |  |
|  | North-East region | 121 912 (26.2) | 29 627 (29.7) | 9 025 (28.3) | 52 690 (26.0) | 24 876 (25.0) | 3 644 (21.0) | 2 050 (13.9) |
|  | North-West region | 74 331 (16.0) | 12 543 (12.6) | 6 648 (20.9) | 32 963 (16.3) | 16 640 (16.7) | 3 409 (19.6) | 2 128 (14.5) |
|  | South-East region | 126 423 (27.1) | 18 974 (19.0) | 7 416 (23.3) | 63 266 (31.2) | 25 500 (25.6) | 4 881 (28.1) | 6 386 (43.4) |
|  | South-West region | 34 205 (7.3) | 5 165 (5.2) | 2 848 (8.9) | 16 618 (8.2) | 6 126 (6.2) | 1 646 (9.5) | 1 802 (12.2) |
|  | Ile-de-France region | 108 879 (23.4) | 33 324 (33.4) | 5 903 (18.5) | 37 234 (18.4) | 26 288 (26.4) | 3 772 (21.7) | 2 358 (16.0) |
| Any pre-existing mental disorder | | 153 870 (33.0) | 35 099 (35.2) | 12 668 (39.8) | 73 923 (36.5) | 25 091 (25.2) | 4 009 (23.1) | 3 080 (20.9) |
|  | Dementia | 67 539 (14.5) | 16 531 (16.6) | 5 264 (16.5) | 34 206 (16.9) | 9 171 (9.2) | 1 330 (7.7) | 1 037 (7.0) |
|  | Depression | 49 420 (10.6) | 11 331 (11.4) | 3 487 (11.0) | 24 321 (12.0) | 8 074 (8.1) | 1 185 (6.8) | 1 022 (6.9) |
|  | Anxiety disorders | 46 039 (9.9) | 10 118 (10.2) | 3 385 (10.6) | 22 934 (11.3) | 7 446 (7.5) | 1 210 (7.0) | 946 (6.4) |
|  | Schizophrenia | 13 400 (2.9) | 3 474 (3.5) | 1 146 (3.6) | 6 102 (3.0) | 2 052 (2.1) | 328 (1.9) | 298 (2.0) |
|  | Alcohol use disorders | 36 509 (7.8) | 8 282 (8.3) | 3 933 (12.4) | 16 163 (8.0) | 6 261 (6.3) | 1 123 (6.5) | 747 (5.1) |
|  | Opioid use disorders | 3 088 (0.7) | 814 (0.8) | 414 (1.3) | 1 258 (0.6) | 418 (0.4) | 109 (0.6) | 75 (0.5) |
|  | Down syndrome | 1 122 (0.2) | 261 (0.3) | 41 (0.1) | 538 (0.3) | 239 (0.2) | 19 (0.1) | 24 (0.2) |
|  | Other learning disabilities | 5 077 (1.1) | 1 247 (1.3) | 381 (1.2) | 2 424 (1.2) | 837 (0.8) | 113 (0.7) | 75 (0.5) |
|  | Other disorder with psychiatric ward admission | 2 869 (0.6) | 622 (0.6) | 220 (0.7) | 1 328 (0.7) | 531 (0.5) | 89 (0.5) | 79 (0.5) |
| Any risk factor for severe COVID-19 | | 337 312 (72.4) | 72 176 (72.4) | 23 586 (74.1) | 152 500 (75.2) | 69 231 (69.6) | 10 857 (62.6) | 8 962 (60.9) |
|  | Tobacco smoking | 57 209 (12.3) | 12 932 (13.0) | 5 745 (18.0) | 24 645 (12.2) | 10 410 (10.5) | 1 967 (11.3) | 1 510 (10.3) |
|  | Obesity (BMI≥30 kg/m^2^) | 117 385 (25.2) | 23 462 (23.5) | 7 304 (22.9) | 51 035 (25.2) | 27 665 (27.8) | 4 428 (25.5) | 3 491 (23.7) |
|  | Hypertension | 270 818 (58.1) | 59 415 (59.6) | 18 903 (59.4) | 126 340 (62.3) | 52 527 (52.8) | 7 482 (43.1) | 6 151 (41.8) |
|  | Diabetes mellitus | 135 162 (29.0) | 28 085 (28.2) | 8 860 (27.8) | 62 240 (30.7) | 28 066 (28.2) | 4 156 (24.0) | 3 755 (25.5) |
| Any severe somatic comorbidity | | 265 912 (57.1) | 58 992 (59.2) | 21 119 (66.3) | 124 110 (61.2) | 47 951 (48.2) | 7 578 (43.7) | 6 162 (41.9) |
|  | Congestive heart failure | 114 419 (24.6) | 25 321 (25.4) | 9 315 (29.3) | 55 515 (27.4) | 19 196 (19.3) | 2 799 (16.1) | 2 273 (15.4) |
|  | Peripheral vascular disease | 54 756 (11.8) | 11 962 (12.0) | 4 540 (14.3) | 26 594 (13.1) | 9 260 (9.3) | 1 328 (7.7) | 1 072 (7.3) |
|  | Cerebrovascular disease | 65 265 (14.0) | 14 613 (14.7) | 5 229 (16.4) | 31 731 (15.6) | 10 697 (10.8) | 1 638 (9.4) | 1 357 (9.2) |
|  | Chronic pulmonary disease | 81 971 (17.6) | 18 630 (18.7) | 6 621 (20.8) | 37 189 (18.3) | 15 174 (15.3) | 2 405 (13.9) | 1 952 (13.3) |
|  | Hemiplegia | 34 189 (7.3) | 7 740 (7.8) | 2 848 (8.9) | 16 179 (8.0) | 5 715 (5.7) | 962 (5.5) | 745 (5.1) |
|  | Moderate or severe liver disease | 7 827 (1.7) | 1 724 (1.7) | 828 (2.6) | 3 462 (1.7) | 1 371 (1.4) | 266 (1.5) | 176 (1.2) |
|  | Mild liver disease | 19 077 (4.1) | 4 210 (4.2) | 1 624 (5.1) | 8 283 (4.1) | 3 847 (3.9) | 618 (3.6) | 495 (3.4) |
|  | Moderate or severe renal disease | 69 394 (14.9) | 15 946 (16.0) | 5 134 (16.1) | 34 049 (16.8) | 11 213 (11.3) | 1 692 (9.8) | 1 360 (9.2) |
|  | Metastatic solid tumour | 26 008 (5.6) | 5 829 (5.9) | 2 816 (8.8) | 12 028 (5.9) | 4 143 (4.2) | 700 (4.0) | 492 (3.3) |
|  | Solid tumour without metastasis | 43 065 (9.2) | 9 326 (9.4) | 3 507 (11.0) | 20 499 (10.1) | 7 677 (7.7) | 1 165 (6.7) | 891 (6.1) |
|  | Haematological malignancy | 15 226 (3.3) | 3 267 (3.3) | 1 439 (4.5) | 7 031 (3.5) | 2 608 (2.6) | 485 (2.8) | 396 (2.7) |
|  | AIDS | 1 802 (0.4) | 572 (0.6) | 177 (0.6) | 589 (0.3) | 343 (0.3) | 66 (0.4) | 55 (0.4) |
|  | Transplant recipient | 8 397 (1.8) | 1 898 (1.9) | 739 (2.3) | 3 661 (1.8) | 1 485 (1.5) | 303 (1.7) | 311 (2.1) |
| Delay between the latest acute hospital discharge and first COVID-19 record | |  |  |  |  |  |  |  |
|  | SARS-CoV-2 infection during hospital care | 70 427 (15.1) | 15 383 (15.4) | 5 998 (18.8) | 35 033 (17.3) | 11 193 (11.3) | 1 709 (9.8) | 1 111 (7.5) |
|  | Previous discharge in the last 3 months | 83 000 (17.8) | 19 092 (19.2) | 6 793 (21.3) | 38 046 (18.8) | 14 693 (14.8) | 2 509 (14.5) | 1 867 (12.7) |
|  | Previous discharge in the last 4-12 months | 92 456 (19.9) | 18 468 (18.5) | 5 512 (17.3) | 41 286 (20.4) | 21 032 (21.2) | 3 251 (18.7) | 2 907 (19.7) |
|  | Previous discharge in the last 2-3 years | 71 909 (15.4) | 16 743 (16.8) | 5 746 (18.0) | 31 506 (15.5) | 13 370 (13.4) | 2 365 (13.6) | 2 179 (14.8) |
|  | Previous discharge in the last 4-9 years | 72 582 (15.6) | 13 982 (14.0) | 3 666 (11.5) | 30 431 (15.0) | 18 543 (18.6) | 3 134 (18.1) | 2 826 (19.2) |
|  | No previous admission in the last 9 years | 75 376 (16.2) | 15 965 (16.0) | 4 125 (13.0) | 26 469 (13.1) | 20 599 (20.7) | 4 384 (25.3) | 3 834 (26.0) |
| Outcomes | |  |  |  |  |  |  |  |
|  | 120-day mortality | 103 890 (22.3) | 23 006 (23.1) | 5 873 (18.4) | 51 382 (25.3) | 18 876 (19.0) | 2 423 (14.0) | 2 330 (15.8) |
|  | Salvage therapy including: | 92 986 (20.0) | 19 276 (19.3) | 5 006 (15.7) | 37 890 (18.7) | 23 278 (23.4) | 3 835 (22.1) | 3 701 (25.1) |
|  | Intensive care unit (ICU) admission or | 81 686 (17.5) | 17 652 (17.7) | 4 541 (14.3) | 33 033 (16.3) | 19 877 (20.0) | 3 392 (19.5) | 3 191 (21.7) |
|  | 1) Extracorporeal membrane oxygenation | 1 287 (0.3) | 342 (0.3) | 33 (0.1) | 428 (0.2) | 352 (0.4) | 63 (0.4) | 69 (0.5) |
|  | 2) Invasive mechanical respiratory support | 53 765 (11.5) | 13 102 (13.2) | 2 817 (8.8) | 21 153 (10.4) | 12 642 (12.7) | 1 998 (11.5) | 2 053 (13.9) |
|  | 3) Continuous positive airway pressure | 24 612 (5.3) | 2 270 (2.3) | 805 (2.5) | 11 019 (5.4) | 7 993 (8.0) | 1 296 (7.5) | 1 229 (8.3) |

# Table B. 120-day mortality risk of inpatients with symptomatic COVID-19 by salvage therapy triage, univariate analyses (n=465 750)

| **Risk factors** | | **Salvage therapy** | | | **No salvage therapy** | | |
| --- | --- | --- | --- | --- | --- | --- | --- |
|  |  | **All patients (%)** | **120-day mortality (%)** | **Censored alive (%)** | **All patients (%)** | **120-day mortality (%)** | **Censored alive (%)** |
|  |  | **92,986 (100.0)** | **27,316 (29.4)** | **65,670 (70.6)** | **372,764 (100.0)** | **76,574 (20.5)** | **296,190 (79.5)** |
| COVID-19 pandemic period | |  |  |  |  |  |  |
|  | First wave (2020 weeks 11-19) | 19,276 (20.7) | 5,673 (20.8) | 13,603 (20.7) | 80,357 (21.6) | 17,333 (22.6) | 63,024 (21.3) |
|  | First inter-wave (2020 weeks 20-36) | 5,006 (5.4) | 1,412 (5.2) | 3,594 (5.5) | 26,834 (7.2) | 4,461 (5.8) | 22,373 (7.6) |
|  | Second wave (2020 week 37-2021 week 8) | 37,890 (40.7) | 12,431 (45.5) | 25,459 (38.8) | 164,881 (44.2) | 38,951 (50.9) | 125,930 (42.5) |
|  | Third wave (2021 weeks 9-19) | 23,278 (25.0) | 6,121 (22.4) | 17,157 (26.1) | 76,152 (20.4) | 12,755 (16.7) | 63,397 (21.4) |
|  | Second inter-wave (2021 weeks 20-30) | 3,835 (4.1) | 839 (3.1) | 2,996 (4.6) | 13,517 (3.6) | 1,584 (2.1) | 11,933 (4.0) |
|  | Fourth wave (2021 weeks 31-34) | 3,701 (4.0) | 840 (3.1) | 2,861 (4.4) | 11,023 (3.0) | 1,490 (1.9) | 9,533 (3.2) |
| COVID-19 with respiratory symptoms | | 82,792 (89.0) | 24,433 (89.4) | 58,359 (88.9) | 288,224 (77.3) | 62,681 (81.9) | 225,543 (76.1) |
| COVID-19-related multisystem inflammatory syndrome | | 25,930 (27.9) | 8,271 (30.3) | 17,659 (26.9) | 84,855 (22.8) | 19,328 (25.2) | 65,527 (22.1) |
| Male | | 61,029 (65.6) | 18,565 (68.0) | 42,464 (64.7) | 190,331 (51.1) | 41,515 (54.2) | 148,816 (50.2) |
| Age, median (IQR) years | | 67 (56-74) | 73 (66-80) | 63 (53-72) | 74 (59-85) | 85 (78-90) | 70 (55-83) |
|  | ≥90 years | 1,787 (1.9) | 1,023 (3.7) | 764 (1.2) | 48,725 (13.1) | 21,238 (27.7) | 27,487 (9.3) |
|  | 85-89 years | 3,900 (4.2) | 2,302 (8.4) | 1,598 (2.4) | 52,948 (14.2) | 19,517 (25.5) | 33,431 (11.3) |
|  | 80-84 years | 6,649 (7.2) | 3,611 (13.2) | 3,038 (4.6) | 45,952 (12.3) | 13,965 (18.2) | 31,987 (10.8) |
|  | 75-79 years | 10,555 (11.4) | 4,783 (17.5) | 5,772 (8.8) | 34,565 (9.3) | 7,519 (9.8) | 27,046 (9.1) |
|  | 70-74 years | 15,172 (16.3) | 5,460 (20.0) | 9,712 (14.8) | 36,830 (9.9) | 5,888 (7.7) | 30,942 (10.4) |
|  | 65-69 years | 14,026 (15.1) | 4,168 (15.3) | 9,858 (15.0) | 30,050 (8.1) | 3,535 (4.6) | 26,515 (9.0) |
|  | 60-64 years | 11,609 (12.5) | 2,600 (9.5) | 9,009 (13.7) | 26,292 (7.1) | 2,129 (2.8) | 24,163 (8.2) |
|  | 55-59 years | 9,265 (10.0) | 1,501 (5.5) | 7,764 (11.8) | 23,990 (6.4) | 1,308 (1.7) | 22,682 (7.7) |
|  | 50-54 years | 6,948 (7.5) | 873 (3.2) | 6,075 (9.3) | 19,388 (5.2) | 720 (0.9) | 18,668 (6.3) |
|  | 45-49 years | 4,623 (5.0) | 444 (1.6) | 4,179 (6.4) | 15,273 (4.1) | 382 (0.5) | 14,891 (5.0) |
|  | 40-44 years | 3,004 (3.2) | 229 (0.8) | 2,775 (4.2) | 10,788 (2.9) | 175 (0.2) | 10,613 (3.6) |
|  | 35-39 years | 2,196 (2.4) | 148 (0.5) | 2,048 (3.1) | 9,242 (2.5) | 93 (0.1) | 9,149 (3.1) |
|  | 30-34 years | 1,503 (1.6) | 81 (0.3) | 1,422 (2.2) | 8,005 (2.1) | 58 (0.1) | 7,947 (2.7) |
|  | 18-29 years | 1,749 (1.9) | 93 (0.3) | 1,656 (2.5) | 10,716 (2.9) | 47 (0.1) | 10,669 (3.6) |
| Area deprivation index quintile | |  |  |  |  |  |  |
|  | FDeP 5 (most deprived) | 23,345 (25.1) | 7,369 (27.0) | 15,976 (24.3) | 91,135 (24.4) | 18,910 (24.7) | 72,225 (24.4) |
|  | FDeP 4 | 17,425 (18.7) | 5,192 (19.0) | 12,233 (18.6) | 75,452 (20.2) | 16,137 (21.1) | 59,315 (20.0) |
|  | FDeP 3 | 17,072 (18.4) | 4,912 (18.0) | 12,160 (18.5) | 68,318 (18.3) | 14,100 (18.4) | 54,218 (18.3) |
|  | FDep 2 | 17,309 (18.6) | 4,822 (17.7) | 12,487 (19.0) | 66,944 (18.0) | 13,353 (17.4) | 53,591 (18.1) |
|  | FDep 1 (least deprived) | 17,835 (19.2) | 5,021 (18.4) | 12,814 (19.5) | 70,915 (19.0) | 14,074 (18.4) | 56,841 (19.2) |
| Residency in metropolitan France | |  |  |  |  |  |  |
|  | North-East region | 23,473 (25.2) | 7,632 (27.9) | 15,841 (24.1) | 98,439 (26.4) | 22,458 (29.3) | 75,981 (25.7) |
|  | North-West region | 12,205 (13.1) | 2,972 (10.9) | 9,233 (14.1) | 62,126 (16.7) | 13,229 (17.3) | 48,897 (16.5) |
|  | South-East region | 25,112 (27.0) | 7,275 (26.6) | 17,837 (27.2) | 101,311 (27.2) | 20,384 (26.6) | 80,927 (27.3) |
|  | South-West region | 6,942 (7.5) | 1,857 (6.8) | 5,085 (7.7) | 27,263 (7.3) | 5,333 (7.0) | 21,930 (7.4) |
|  | Ile-de-France region | 25,254 (27.2) | 7,580 (27.7) | 17,674 (26.9) | 83,625 (22.4) | 15,170 (19.8) | 68,455 (23.1) |
| Any pre-existing mental disorder | | 21,500 (23.1) | 7,510 (27.5) | 13,990 (21.3) | 132,370 (35.5) | 39,473 (51.5) | 92,897 (31.4) |
|  | Dementia | 3,836 (4.1) | 1,601 (5.9) | 2,235 (3.4) | 63,703 (17.1) | 22,433 (29.3) | 41,270 (13.9) |
|  | Depression | 6,110 (6.6) | 2,222 (8.1) | 3,888 (5.9) | 43,310 (11.6) | 13,051 (17.0) | 30,259 (10.2) |
|  | Anxiety disorders | 5,342 (5.7) | 2,004 (7.3) | 3,338 (5.1) | 40,697 (10.9) | 12,607 (16.5) | 28,090 (9.5) |
|  | Schizophrenia | 2,409 (2.6) | 607 (2.2) | 1,802 (2.7) | 10,991 (2.9) | 2,542 (3.3) | 8,449 (2.9) |
|  | Alcohol use disorders | 8,681 (9.3) | 2,941 (10.8) | 5,740 (8.7) | 27,828 (7.5) | 6,754 (8.8) | 21,074 (7.1) |
|  | Opioid use disorders | 698 (0.8) | 164 (0.6) | 534 (0.8) | 2,390 (0.6) | 383 (0.5) | 2,007 (0.7) |
|  | Down syndrome | 242 (0.3) | 50 (0.2) | 192 (0.3) | 880 (0.2) | 170 (0.2) | 710 (0.2) |
|  | Other learning disability | 916 (1.0) | 232 (0.8) | 684 (1.0) | 4,161 (1.1) | 716 (0.9) | 3,445 (1.2) |
|  | Other disorders with psychiatric ward admission | 439 (0.5) | 183 (0.7) | 256 (0.4) | 2,430 (0.7) | 775 (1.0) | 1,655 (0.6) |
| Any risk factor for severe COVID-19 | | 72,338 (77.8) | 22,266 (81.5) | 50,072 (76.2) | 264,974 (71.1) | 62,493 (81.6) | 202,481 (68.4) |
|  | Tobacco smoking | 15,458 (16.6) | 4,764 (17.4) | 10,694 (16.3) | 41,751 (11.2) | 8,674 (11.3) | 33,077 (11.2) |
|  | Obesity (BMI≥30 kg/m^2^) | 33,394 (35.9) | 8,690 (31.8) | 24,704 (37.6) | 83,991 (22.5) | 14,888 (19.4) | 69,103 (23.3) |
|  | Hypertension | 54,874 (59.0) | 18,662 (68.3) | 36,212 (55.1) | 215,944 (57.9) | 56,696 (74.0) | 159,248 (53.8) |
|  | Diabetes mellitus | 31,968 (34.4) | 10,439 (38.2) | 21,529 (32.8) | 103,194 (27.7) | 24,052 (31.4) | 79,142 (26.7) |
| Any severe somatic comorbidity | | 51,283 (55.2) | 19,831 (72.6) | 31,452 (47.9) | 214,629 (57.6) | 63,133 (82.4) | 151,496 (51.1) |
|  | Congestive heart failure | 20,774 (22.3) | 9,174 (33.6) | 11,600 (17.7) | 93,645 (25.1) | 33,875 (44.2) | 59,770 (20.2) |
|  | Peripheral vascular disease | 10,599 (11.4) | 4,752 (17.4) | 5,847 (8.9) | 44,157 (11.8) | 14,971 (19.6) | 29,186 (9.9) |
|  | Cerebrovascular disease | 9,883 (10.6) | 4,438 (16.2) | 5,445 (8.3) | 55,382 (14.9) | 18,579 (24.3) | 36,803 (12.4) |
|  | Chronic pulmonary disease | 17,253 (18.6) | 6,440 (23.6) | 10,813 (16.5) | 64,718 (17.4) | 16,566 (21.6) | 48,152 (16.3) |
|  | Hemiplegia | 6,425 (6.9) | 2,272 (8.3) | 4,153 (6.3) | 27,764 (7.4) | 9,094 (11.9) | 18,670 (6.3) |
|  | Moderate or severe liver disease | 1,990 (2.1) | 1,012 (3.7) | 978 (1.5) | 5,837 (1.6) | 2,034 (2.7) | 3,803 (1.3) |
|  | Mild liver disease | 4,683 (5.0) | 1,578 (5.8) | 3,105 (4.7) | 14,394 (3.9) | 2,937 (3.8) | 11,457 (3.9) |
|  | Moderate or severe renal disease | 11,799 (12.7) | 5,756 (21.1) | 6,043 (9.2) | 57,595 (15.5) | 21,287 (27.8) | 36,308 (12.3) |
|  | Metastatic solid tumour | 3,886 (4.2) | 2,133 (7.8) | 1,753 (2.7) | 22,122 (5.9) | 11,098 (14.5) | 11,024 (3.7) |
|  | Solid tumour without metastasis | 8,012 (8.6) | 3,308 (12.1) | 4,704 (7.2) | 35,053 (9.4) | 10,355 (13.5) | 24,698 (8.3) |
|  | Haematological malignancy | 3,471 (3.7) | 1,756 (6.4) | 1,715 (2.6) | 11,755 (3.2) | 4,288 (5.6) | 7,467 (2.5) |
|  | AIDS | 505 (0.5) | 134 (0.5) | 371 (0.6) | 1,297 (0.3) | 131 (0.2) | 1,166 (0.4) |
|  | Transplant recipient | 2,540 (2.7) | 1,068 (3.9) | 1,472 (2.2) | 5,857 (1.6) | 1,041 (1.4) | 4,816 (1.6) |
| Delay between the latest acute hospital discharge and first COVID-19 record | |  |  |  |  |  |  |
|  | SARS-CoV-2 infection during hospital care | 16,894 (18.2) | 6,673 (24.4) | 10,221 (15.6) | 53,533 (14.4) | 19,041 (24.9) | 34,492 (11.6) |
|  | Previous discharge in the last 3 months | 13,089 (14.1) | 5,363 (19.6) | 7,726 (11.8) | 69,911 (18.8) | 20,167 (26.3) | 49,744 (16.8) |
|  | Previous discharge in the last 4-12 months | 17,239 (18.5) | 4,764 (17.4) | 12,475 (19.0) | 75,217 (20.2) | 13,599 (17.8) | 61,618 (20.8) |
|  | Previous discharge in the last 2-3 years | 12,539 (13.5) | 3,950 (14.5) | 8,589 (13.1) | 59,370 (15.9) | 11,941 (15.6) | 47,429 (16.0) |
|  | Previous discharge in the last 4-9 years | 15,248 (16.4) | 3,532 (12.9) | 11,716 (17.8) | 57,334 (15.4) | 8,220 (10.7) | 49,114 (16.6) |
|  | No previous admission in the last 9 years | 17,977 (19.3) | 3,034 (11.1) | 14,943 (22.8) | 57,399 (15.4) | 3,606 (4.7) | 53,793 (18.2) |

# Table C. 120-day mortality risk of inpatients with symptomatic COVID-19 by salvage therapy triage, simultaneous probit multivariate model (n=465 750)

| **Risk factors** | | **Salvage therapy**  **92 986 (20.0%) of 465 750** | | | **120-day mortality with salvage therapy**  **27 316 (29.4%) of 92 986** | | | **120-day mortality without salvage therapy 76 574 (20.5%) of 372 764** | | |
| --- | --- | --- | --- | --- | --- | --- | --- | --- | --- | --- |
|  |  | **Estimate** | **Standard error** | **p-value** | **Estimate** | **Standard error** | **p-value** | **Estimate** | **Standard error** | **p-value** |
| Constant | | -1.604 | 0.019 | <0.001 | -0.468 | 0.093 | <0.001 | -2.207 | 0.043 | <0.001 |
| COVID-19 pandemic period (ref: first inter-wave) | |  |  |  |  |  |  |  |  |  |
|  | First wave (2020 weeks 11-19) | 0.010 | 0.010 | 0.32 | 0.097 | 0.020 | <0.001 | 0.249 | 0.010 | <0.001 |
|  | Second wave (2020 week 37-2021 week 8) | 0.059 | 0.010 | <0.001 | 0.081 | 0.019 | <0.001 | 0.243 | 0.009 | <0.001 |
|  | Third wave (2021 weeks 9-19) | 0.121 | 0.010 | <0.001 | 0.069 | 0.021 | <0.001 | 0.260 | 0.010 | <0.001 |
|  | Second inter-wave (2021 weeks 20-30) | 0.123 | 0.014 | <0.001 | 0.026 | 0.028 | 0.35 | 0.159 | 0.016 | <0.001 |
|  | Fourth wave (2021 weeks 31-34) | 0.221 | 0.015 | <0.001 | 0.045 | 0.029 | 0.12 | 0.310 | 0.017 | <0.001 |
| COVID-19 with respiratory symptoms | | 0.414 | 0.009 | <0.001 |  |  |  |  |  |  |
| COVID-19-related multisystem inflammatory syndrome | | 0.290 | 0.007 | <0.001 |  |  |  |  |  |  |
| Male | | 0.250 | 0.005 | <0.001 | **-0.032** | **0.012** | **0.006** | 0.261 | 0.005 | <0.001 |
| Age category (ref: 18-29 years) | |  |  |  |  |  |  |  |  |  |
|  | ≥90 years | -0.673 | 0.019 | <0.001 | 1.826 | 0.053 | <0.001 | 1.517 | 0.040 | <0.001 |
|  | 85-89 years | -0.403 | 0.018 | <0.001 | 1.664 | 0.052 | <0.001 | 1.354 | 0.040 | <0.001 |
|  | 80-84 years | -0.106 | 0.017 | <0.001 | 1.395 | 0.053 | <0.001 | 1.259 | 0.040 | <0.001 |
|  | 75-79 years | 0.260 | 0.017 | <0.001 | 0.989 | 0.054 | <0.001 | 1.190 | 0.038 | <0.001 |
|  | 70-74 years | 0.414 | 0.016 | <0.001 | 0.714 | 0.054 | <0.001 | 1.115 | 0.037 | <0.001 |
|  | 65-69 years | 0.476 | 0.016 | <0.001 | 0.538 | 0.053 | <0.001 | 1.021 | 0.037 | <0.001 |
|  | 60-64 years | 0.439 | 0.016 | <0.001 | 0.379 | 0.050 | <0.001 | 0.881 | 0.037 | <0.001 |
|  | 55-59 years | 0.359 | 0.016 | <0.001 | 0.253 | 0.048 | <0.001 | 0.733 | 0.037 | <0.001 |
|  | 50-54 years | 0.315 | 0.017 | <0.001 | 0.164 | 0.047 | <0.001 | 0.608 | 0.037 | <0.001 |
|  | 45-49 years | 0.220 | 0.018 | <0.001 | 0.095 | 0.048 | 0.047 | 0.482 | 0.039 | <0.001 |
|  | 40-44 years | 0.179 | 0.019 | <0.001 | 0.017 | 0.051 | 0.74 | 0.367 | 0.042 | <0.001 |
|  | 35-39 years | 0.117 | 0.020 | <0.001 | 0.011 | 0.054 | 0.84 | 0.240 | 0.045 | <0.001 |
|  | 30-34 years | 0.022 | 0.021 | 0.31 | -0.013 | 0.060 | 0.83 | 0.109 | 0.049 | 0.027 |
| Area deprivation index quintile (ref : least deprived) | |  |  |  |  |  |  |  |  |  |
|  | FDeP 5 (most deprived) | 0.010 | 0.007 | 0.17 | 0.160 | 0.013 | <0.001 | 0.078 | 0.008 | <0.001 |
|  | FDeP 4 | -0.016 | 0.007 | 0.030 | 0.123 | 0.014 | <0.001 | 0.055 | 0.008 | <0.001 |
|  | FDeP 3 | 0.024 | 0.007 | 0.002 | 0.079 | 0.014 | <0.001 | 0.066 | 0.008 | <0.001 |
|  | FDep 2 | 0.016 | 0.007 | 0.024 | 0.068 | 0.013 | <0.001 | 0.056 | 0.008 | <0.001 |
| Residency in metropolitan France (ref: Ile-de-France) | |  |  |  |  |  |  |  |  |  |
|  | North-East region | -0.101 | 0.007 | <0.001 | -0.047 | 0.013 | <0.001 | -0.024 | 0.007 | <0.001 |
|  | North-West region | -0.180 | 0.008 | <0.001 | -0.184 | 0.018 | <0.001 | -0.130 | 0.008 | <0.001 |
|  | South-East region | -0.059 | 0.006 | <0.001 | -0.136 | 0.012 | <0.001 | -0.077 | 0.007 | <0.001 |
|  | South-West region | -0.001 | 0.009 | 0.94 | -0.245 | 0.018 | <0.001 | -0.107 | 0.010 | <0.001 |
| Any pre-existing mental disorder | |  |  |  |  |  |  |  |  |  |
|  | Dementia | -0.489 | 0.009 | <0.001 | 0.083 | 0.024 | <0.001 | **-0.048** | **0.006** | **<0.001** |
|  | Depression | -0.103 | 0.010 | <0.001 | 0.080 | 0.019 | <0.001 | 0.020 | 0.008 | 0.013 |
|  | Anxiety disorders | -0.086 | 0.010 | <0.001 | 0.053 | 0.019 | 0.007 | 0.003 | 0.008 | 0.70 |
|  | Schizophrenia | -0.078 | 0.014 | <0.001 | 0.034 | 0.026 | 0.19 | 0.109 | 0.013 | <0.001 |
|  | Alcohol use disorders | -0.088 | 0.009 | <0.001 | 0.089 | 0.015 | <0.001 | 0.051 | 0.009 | <0.001 |
|  | Opioid use disorders | 0.059 | 0.026 | 0.023 | -0.041 | 0.048 | 0.40 | 0.009 | 0.029 | 0.75 |
|  | Down syndrome | -0.014 | 0.042 | 0.74 | 0.427 | 0.080 | <0.001 | 0.730 | 0.043 | <0.001 |
|  | Other learning disabilities | -0.201 | 0.021 | <0.001 | 0.341 | 0.040 | <0.001 | 0.230 | 0.021 | <0.001 |
|  | Other disorder with psychiatric ward admission | -0.138 | 0.029 | <0.001 | 0.172 | 0.055 | 0.002 | 0.098 | 0.026 | <0.001 |
| Any risk factor for severe COVID-19 | |  |  |  |  |  |  |  |  |  |
|  | Tobacco smoking | 0.103 | 0.007 | <0.001 | **-0.114** | **0.012** | **<0.001** | 0.049 | 0.008 | <0.001 |
|  | Obesity (BMI≥30 kg/m^2^) | 0.249 | 0.005 | <0.001 | **-0.176** | **0.010** | **<0.001** | 0.074 | 0.006 | <0.001 |
|  | Hypertension | 0.141 | 0.005 | <0.001 | -0.163 | 0.010 | <0.001 | -0.037 | 0.006 | <0.001 |
|  | Diabetes mellitus | 0.038 | 0.005 | <0.001 | **-0.024** | **0.009** | **0.007** | 0.040 | 0.005 | <0.001 |
| Any severe somatic comorbidity | |  |  |  |  |  |  |  |  |  |
|  | Congestive heart failure | 0.079 | 0.006 | <0.001 | 0.079 | 0.011 | <0.001 | 0.203 | 0.006 | <0.001 |
|  | Peripheral vascular disease | -0.057 | 0.008 | <0.001 | 0.084 | 0.013 | <0.001 | 0.035 | 0.007 | <0.001 |
|  | Cerebrovascular disease | -0.089 | 0.008 | <0.001 | 0.199 | 0.013 | <0.001 | 0.006 | 0.007 | 0.37 |
|  | Chronic pulmonary disease | -0.011 | 0.006 | 0.060 | 0.086 | 0.010 | <0.001 | 0.012 | 0.006 | 0.038 |
|  | Hemiplegia | -0.014 | 0.009 | 0.12 | 0.001 | 0.016 | 0.95 | 0.178 | 0.009 | <0.001 |
|  | Moderate or severe liver disease | 0.025 | 0.017 | 0.13 | 0.498 | 0.029 | <0.001 | 0.374 | 0.016 | <0.001 |
|  | Mild liver disease | -0.007 | 0.011 | 0.49 | 0.121 | 0.018 | <0.001 | 0.051 | 0.011 | <0.001 |
|  | Moderate or severe renal disease | -0.029 | 0.007 | <0.001 | 0.203 | 0.013 | <0.001 | 0.116 | 0.006 | <0.001 |
|  | Metastatic solid tumour | -0.348 | 0.010 | <0.001 | 0.668 | 0.019 | <0.001 | 0.676 | 0.011 | <0.001 |
|  | Solid tumour without metastasis | -0.074 | 0.008 | <0.001 | 0.114 | 0.014 | <0.001 | 0.108 | 0.007 | <0.001 |
|  | Haematological malignancy | 0.092 | 0.012 | <0.001 | 0.266 | 0.022 | <0.001 | 0.333 | 0.011 | <0.001 |
|  | AIDS | 0.046 | 0.032 | 0.16 | -0.011 | 0.055 | 0.84 | 0.020 | 0.041 | 0.63 |
|  | Transplant recipient | 0.155 | 0.016 | <0.001 | 0.067 | 0.025 | 0.008 | 0.043 | 0.017 | 0.012 |
| Delay between the latest acute hospital discharge and first COVID-19 record (ref: no previous admission in the last 9 years) | |  |  |  |  |  |  |  |  |  |
|  | SARS-CoV-2 infection during hospital care | 0.200 | 0.008 | <0.001 | 0.131 | 0.017 | <0.001 | 0.337 | 0.010 | <0.001 |
|  | Previous discharge in the last 3 months | -0.180 | 0.008 | <0.001 | 0.298 | 0.015 | <0.001 | 0.101 | 0.010 | <0.001 |
|  | Previous discharge in the last 4-12 months | -0.121 | 0.008 | <0.001 | 0.146 | 0.015 | <0.001 | -0.022 | 0.010 | 0.031 |
|  | Previous discharge in the last 2-3 years | -0.092 | 0.007 | <0.001 | 0.086 | 0.014 | <0.001 | -0.002 | 0.010 | 0.81 |
|  | Previous discharge in the last 4-9 years | -0.045 | 0.008 | <0.001 | 0.031 | 0.014 | 0.031 | 0.014 | 0.010 | 0.17 |
| Unobserved heterogeneity (rho correlation) | |  |  |  |  |  |  |  |  |  |
|  | Salvage therapy and mortality with salvage therapy |  |  |  | -0.667 | 0.025 | <0.001 |  |  |  |
|  | No salvage therapy and mortality without salvage therapy |  |  |  |  |  |  | -0.918 | 0.006 | <0.001 |

Legend: To further assess the independent effects of the risk factors on all-cause mortality depending on salvage therapy triage, we estimated a simultaneous probit multivariate model with three dependent variables (salvage therapy, mortality with salvage therapy, and mortality without salvage therapy) and joint estimation to control for unobserved heterogeneity and omitted variable bias.[1, 2] Two covariates (COVID-19 with respiratory symptoms, COVID-19-related multisystem inflammatory syndrome) were maintained in the salvage therapy equation for identification purposes.

The direction of associations with mortality risk was similar with or without salvage therapy for most covariates including almost all mental disorders. The direction was significantly inverted for five covariates (in red): dementia (i.e., higher mortality risk with salvage therapy vs. lower mortality risk without salvage therapy) and male gender, tobacco smoking, obesity, and diabetes mellitus (i.e., lower mortality risk with salvage therapy vs. higher mortality risk without salvage therapy), suggesting that these criteria were associated with selection biases in salvage therapy triage.

After taking all covariates and unobserved heterogeneity into account, rho correlation coefficient estimates were strongly negative, suggesting that the remaining mortality risk would be very low in patients without salvage therapy (rho=-0.92; p<0.001) and low in patients with salvage therapy (rho=-0.67; p<0.001).

Reference

1. Chib S, Geenberg E. Analysis of multivariate probit models. Biometrika. 1998;85(2):347–61.

2. Costa-Font J, Gil J. Obesity and the incidence of chronic diseases in Spain: a seemingly unrelated probit approach. Economics and human biology. 2005;3(2):188-214.

# Table D. Controlled direct effects of pre-existing mental disorders on 120-day mortality risk of inpatients with symptomatic COVID-19, causal mediation analyses (n=465 750)

| Category of pre-existing mental disorder | Total effect | | Natural direct effect without salvage therapy | | Natural indirect effect following salvage therapy | | Controlled direct effect  without salvage therapy, i.e. direct effect that is not due to mediation or interaction  with salvage therapy | | Controlled direct effect  with salvage therapy | |
| --- | --- | --- | --- | --- | --- | --- | --- | --- | --- | --- |
|  | Adjusted odds-ratio (95% CI) | p-value | Adjusted odds-ratio (95% CI) | p-value | Adjusted odds-ratio (95% CI) | p-value | Adjusted odds-ratio (95% CI) | p-value | Adjusted odds-ratio (95% CI) | p-value |
| Dementia | 0.88 (0.86-0.90) | <0.001 | 0.94 (0.91-0.96) | <0.001 | 0.94 (0.93-0.95) | <0.001 | 1.17 (1.14-1.19) | <0.001 | 0.60 (0.56-0.65) | <0.001 |
| Depression | 1.03 (0.99-1.06) | 0.088 | 1.06 (1.03-1.09) | <0.001 | 0.97 (0.96-0.97) | <0.001 | 1.13 (1.09-1.16) | <0.001 | 0.95 (0.89-1.01) | 0.13 |
| Anxiety disorders | 0.98 (0.95-1.01) | 0.29 | 1.01 (0.98-1.04) | 0.54 | 0.97 (0.97-0.98) | <0.001 | 1.08 (1.05-1.11) | <0.001 | 0.89 (0.83-0.95) | <0.001 |
| Schizophrenia | 1.14 (1.09-1.20) | <0.001 | 1.15 (1.10-1.21) | <0.001 | 0.99 (0.98-0,99) | 0.005 | 1.29 (1.23-1.36) | <0.001 | 0.91 (0.82-1.01) | 0.064 |
| Alcohol use disorders | 1.17 (1.13-1.21) | <0.001 | 1.19 (1.15-1.23) | <0.001 | 0.98 (0.97-0.99) | <0.001 | 1.26 (1.21-1.30) | <0.001 | 1.08 (1.02-1.14) | 0.008 |
| Opioid use disorders | 0.99 (0.88-1.10) | 0.80 | 0.97 (0.86-1.07) | 0.52 | 1.02 (1.00-1.04) | 0.040 | 0.99 (0.87-1.12) | 0.89 | 0.92 (0.73-1.11) | 0.40 |
| Down syndrome | 5.11 (4.30-5.91) | <0.001 | 5.11 (4.30-5.91) | <0.001 | 1.00 (0.99-1.01) | 0.97 | 6.34 (5.20-7.47) | <0.001 | 2.96 (1.98-3.94) | <0.001 |
| Other learning disabilities | 1.86 (1.72-2.01) | <0.001 | 1.94 (1.79-2.10) | <0.001 | 0.96 (0.95-0.97) | <0.001 | 2.15 (1.95-2.34) | <0.001 | 1.58 (1.32-1.84) | <0.001 |
| Other disorder with psychiatric ward admission | 1.27 (1.15-1.40) | <0.001 | 1.33 (1.20-1.47) | <0.001 | 0.96 (0.93-0.98) | <0.001 | 1.34 (1.21-1.47) | <0.001 | 1.32 (1.02-1.61) | 0.035 |

Legend: Causal mediation effects on mortality risk were assessed per category of pre-existing mental disorder in a counterfactual framework with an interaction term between exposure (category of pre-existing mental disorder) and mediator (access to salvage therapy) and control for all other prognostic factors (including all other categories of mental disorders) [1, 2]. The “natural direct effect” (without salvage therapy) includes two component effects: the “controlled direct effect” (without salvage therapy) that is not due to mediation or interaction with salvage therapy and the interaction effect that is due to interaction but not mediation.

Reference

1. Pearl J. Causality: Models, Reasoning, and Inferences. 2nd edition. Cambridge: Cambridge University Press; 2009.

2. Valeri L, Vanderweele TJ. Mediation analysis allowing for exposure-mediator interactions and causal interpretation: theoretical assumptions and implementation with SAS and SPSS macros. Psychol Methods. 2013;18(2):137-50.

# Table E. Characteristics of inpatients with symptomatic COVID-19 by pre-existing mental disorders (n=465 750)

| **Risk factors and outcomes** | | **All patients (%)** | **Any pre-existing**  **mental disorder (%)** | **No mental disorder (%)** |
| --- | --- | --- | --- | --- |
|  |  | **465 750 (100.0)** | **153 870 (33.0)** | **311 880 (67.0)** |
| COVID-19 pandemic period | |  |  |  |
|  | First wave (2020 weeks 11-19) | 99 633 (21.4) | 35 099 (22.8) | 64 534 (20.7) |
|  | First inter-wave (2020 weeks 20-36) | 31 840 (6.8) | 12 668 (8.2) | 19 172 (6.1) |
|  | Second wave (2020 week 37-2021 week 8) | 202 771 (43.5) | 73 923 (48.0) | 128 848 (41.3) |
|  | Third wave (2021 weeks 9-19) | 99 430 (21.3) | 25 091 (16.3) | 74 339 (23.8) |
|  | Second inter-wave (2021 weeks 20-30) | 17 352 (3.7) | 4 009 (2.6) | 13 343 (4.3) |
|  | Fourth wave (2021 weeks 31-34) | 14 724 (3.2) | 3 080 (2.0) | 11 644 (3.7) |
| COVID-19 with respiratory symptoms | | 371 016 (79.7) | 114 476 (74.4) | 256 540 (82.3) |
| COVID-19-related multisystem inflammatory syndrome | | 110 785 (23.8) | 41 284 (26.8) | 69 501 (22.3) |
| Male | | 251 360 (54.0) | 75 425 (49.0) | 175 935 (56.4) |
| Age, median (IQR) years | | 72 (58-84) | 80 (67-88) | 68 (55-80) |
|  | ≥90 years | 50 512 (10.8) | 27 379 (17.8) | 23 133 (7.4) |
|  | 85-89 years | 56 848 (12.2) | 28 104 (18.3) | 28 744 (9.2) |
|  | 80-84 years | 52 601 (11.3) | 22 618 (14.7) | 29 983 (9.6) |
|  | 75-79 years | 45 120 (9.7) | 15 710 (10.2) | 29 410 (9.4) |
|  | 70-74 years | 52 002 (11.2) | 15 368 (10.0) | 36 634 (11.7) |
|  | 65-69 years | 44 076 (9.5) | 12 216 (7.9) | 31 860 (10.2) |
|  | 60-64 years | 37 901 (8.1) | 9 520 (6.2) | 28 381 (9.1) |
|  | 55-59 years | 33 255 (7.1) | 7 298 (4.7) | 25 957 (8.3) |
|  | 50-54 years | 26 336 (5.7) | 5 268 (3.4) | 21 068 (6.8) |
|  | 45-49 years | 19 896 (4.3) | 3 548 (2.3) | 16 348 (5.2) |
|  | 40-44 years | 13 792 (3.0) | 2 224 (1.4) | 11 568 (3.7) |
|  | 35-39 years | 11 438 (2.5) | 1 770 (1.2) | 9 668 (3.1) |
|  | 30-34 years | 9 508 (2.0) | 1 339 (0.9) | 8 169 (2.6) |
|  | 18-29 years | 12 465 (2.7) | 1 508 (1.0) | 10 957 (3.5) |
| Area deprivation index quintile | |  |  |  |
|  | FDeP 5 (most deprived) | 114 480 (24.6) | 37 730 (24.5) | 76 750 (24.6) |
|  | FDeP 4 | 92 877 (19.9) | 32 103 (20.9) | 60 774 (19.5) |
|  | FDeP 3 | 85 390 (18.3) | 27 903 (18.1) | 57 487 (18.4) |
|  | FDep 2 | 84 253 (18.1) | 26 927 (17.5) | 57 326 (18.4) |
|  | FDep 1 (least deprived) | 88 750 (19.1) | 29 207 (19.0) | 59 543 (19.1) |
| Residency in metropolitan France | |  |  |  |
|  | North-East region | 121 912 (26.2) | 43 854 (28.5) | 78 058 (25.0) |
|  | North-West region | 74 331 (16.0) | 26 685 (17.3) | 47 646 (15.3) |
|  | South-East region | 126 423 (27.1) | 40 407 (26.3) | 86 016 (27.6) |
|  | South-West region | 34 205 (7.3) | 12 126 (7.9) | 22 079 (7.1) |
|  | Ile-de-France region | 108 879 (23.4) | 30 798 (20.0) | 78 081 (25.0) |
| Any pre-existing mental disorder | | 153 870 (33.0) | 153 870 (100.0) |  |
|  | Dementia | 67 539 (14.5) | 67 539 (43.9) |  |
|  | Depression | 49 420 (10.6) | 49 420 (32.1) |  |
|  | Anxiety disorders | 46 039 (9.9) | 46 039 (29.9) |  |
|  | Schizophrenia | 13 400 (2.9) | 13 400 (8.7) |  |
|  | Alcohol use disorders | 36 509 (7.8) | 36 509 (23.7) |  |
|  | Opioid use disorders | 3 088 (0.7) | 3 088 (2.0) |  |
|  | Down syndrome | 1 122 (0.2) | 1 122 (0.7) |  |
|  | Other learning disabilities | 5 077 (1.1) | 5 077 (3.3) |  |
|  | Other disorder with psychiatric ward admission | 2 869 (0.6) | 2 869 (1.9) |  |
| Any risk factor for severe COVID-19 | | 337 312 (72.4) | 128 084 (83.2) | 209 228 (67.1) |
|  | Tobacco smoking | 57 209 (12.3) | 29 591 (19.2) | 27 618 (8.9) |
|  | Obesity (BMI≥30 kg/m^2^) | 117 385 (25.2) | 40 129 (26.1) | 77 256 (24.8) |
|  | Hypertension | 270 818 (58.1) | 109 376 (71.1) | 161 442 (51.8) |
|  | Diabetes mellitus | 135 162 (29.0) | 48 739 (31.7) | 86 423 (27.7) |
| Any severe somatic comorbidity | | 265 912 (57.1) | 114 608 (74.5) | 151 304 (48.5) |
|  | Congestive heart failure | 114 419 (24.6) | 54 327 (35.3) | 60 092 (19.3) |
|  | Peripheral vascular disease | 54 756 (11.8) | 26 815 (17.4) | 27 941 (9.0) |
|  | Cerebrovascular disease | 65 265 (14.0) | 37 099 (24.1) | 28 166 (9.0) |
|  | Chronic pulmonary disease | 81 971 (17.6) | 36 017 (23.4) | 45 954 (14.7) |
|  | Hemiplegia | 34 189 (7.3) | 18 516 (12.0) | 15 673 (5.0) |
|  | Moderate or severe liver disease | 7 827 (1.7) | 5 682 (3.7) | 2 145 (0.7) |
|  | Mild liver disease | 19 077 (4.1) | 10 097 (6.6) | 8 980 (2.9) |
|  | Moderate or severe renal disease | 69 394 (14.9) | 34 089 (22.2) | 35 305 (11.3) |
|  | Metastatic solid tumour | 26 008 (5.6) | 9 851 (6.4) | 16 157 (5.2) |
|  | Solid tumour without metastasis | 43 065 (9.2) | 18 435 (12.0) | 24 630 (7.9) |
|  | Haematological malignancy | 15 226 (3.3) | 5 288 (3.4) | 9 938 (3.2) |
|  | AIDS | 1 802 (0.4) | 608 (0.4) | 1 194 (0.4) |
|  | Transplant recipient | 8 397 (1.8) | 3 080 (2.0) | 5 317 (1.7) |
| Delay between the latest acute hospital discharge and first COVID-19 record | |  |  |  |
|  | SARS-CoV-2 infection during hospital care | 70 427 (15.1) | 35 386 (23.0) | 35 041 (11.2) |
|  | Previous discharge in the last 3 months | 83 000 (17.8) | 35 585 (23.1) | 47 415 (15.2) |
|  | Previous discharge in the last 4-12 months | 92 456 (19.9) | 30 821 (20.0) | 61 635 (19.8) |
|  | Previous discharge in the last 2-3 years | 71 909 (15.4) | 29 492 (19.2) | 42 417 (13.6) |
|  | Previous discharge in the last 4-9 years | 72 582 (15.6) | 16 618 (10.8) | 55 964 (17.9) |
|  | No previous admission in the last 9 years | 75 376 (16.2) | 5 968 (3.9) | 69 408 (22.3) |
| Outcomes | |  |  |  |
|  | 120-day mortality | 103 890 (22.3) | 46 983 (30.5) | 56 907 (18.2) |
|  | Salvage therapy including | 92 986 (20.0) | 21 500 (14.0) | 71 486 (22.9) |
|  | Intensive care unit (ICU) admission or | 81 686 (17.5) | 18 390 (12.0) | 63 296 (20.3) |
|  | 1) Extracorporeal membrane oxygenation | 1 287 (0.3) | 139 (0.1) | 1 148 (0.4) |
|  | 2) Invasive mechanical respiratory support | 53 765 (11.5) | 12 617 (8.2) | 41 148 (13.2) |
|  | 3) Continuous positive airway pressure | 24 612 (5.3) | 4 868 (3.2) | 19 744 (6.3) |

# Table F. Characteristics of inpatients with symptomatic COVID-19 by age category and pre-existing mental disorders (n=465 750)

| **Risk factors and outcomes** | | **Patients aged 18-64 years** | | | **Patients aged 65 years or more** | | |
| --- | --- | --- | --- | --- | --- | --- | --- |
|  |  | **All patients (%)** | **Any pre-existing**  **mental disorder (%)** | **No mental disorder (%)** | **All patients (%)** | **Any pre-existing**  **mental disorder (%)** | **No mental disorder (%)** |
|  |  | **164,591 (100.0)** | **32,475 (19.7)** | **132,116 (80.3)** | **301,159 (100.0)** | **121,395 (40.3)** | **179,764 (59.7)** |
| COVID-19 pandemic period | |  |  |  |  |  |  |
|  | First wave (2020 weeks 11-19) | 35,977 (21.9) | 7,593 (23.4) | 28,384 (21.5) | 63,656 (21.1) | 27,506 (22.7) | 36,150 (20.1) |
|  | First inter-wave (2020 weeks 20-36) | 10,917 (6.6) | 3,282 (10.1) | 7,635 (5.8) | 20,923 (6.9) | 9,386 (7.7) | 11,537 (6.4) |
|  | Second wave (2020 week 37-2021 week 8) | 57,642 (35.0) | 12,764 (39.3) | 44,878 (34.0) | 145,129 (48.2) | 61,159 (50.4) | 83,970 (46.7) |
|  | Third wave (2021 weeks 9-19) | 42,618 (25.9) | 6,524 (20.1) | 36,094 (27.3) | 56,812 (18.9) | 18,567 (15.3) | 38,245 (21.3) |
|  | Second inter-wave (2021 weeks 20-30) | 9,539 (5.8) | 1,368 (4.2) | 8,171 (6.2) | 7,813 (2.6) | 2,641 (2.2) | 5,172 (2.9) |
|  | Fourth wave (2021 weeks 31-34) | 7,898 (4.8) | 944 (2.9) | 6,954 (5.3) | 6,826 (2.3) | 2,136 (1.8) | 4,690 (2.6) |
| COVID-19 with respiratory symptoms | | 133,418 (81.1) | 22,918 (70.6) | 110,500 (83.6) | 237,598 (78.9) | 91,558 (75.4) | 146,040 (81.2) |
| COVID-19-related multisystem inflammatory syndrome | | 37,399 (22.7) | 10,694 (32.9) | 26,705 (20.2) | 73,386 (24.4) | 30,590 (25.2) | 42,796 (23.8) |
| Male | | 94,949 (57.7) | 19,294 (59.4) | 75,655 (57.3) | 156,411 (51.9) | 56,131 (46.2) | 100,280 (55.8) |
| Age, median (IQR) years | | 53 (42-59) | 55 (47-60) | 52 (41-59) | 80 (73-87) | 83 (75-89) | 78 (71-86) |
|  | ≥90 years |  |  |  | 50,512 (16.8) | 27,379 (22.6) | 23,133 (12.9) |
|  | 85-89 years |  |  |  | 56,848 (18.9) | 28,104 (23.2) | 28,744 (16.0) |
|  | 80-84 years |  |  |  | 52,601 (17.5) | 22,618 (18.6) | 29,983 (16.7) |
|  | 75-79 years |  |  |  | 45,120 (15.0) | 15,710 (12.9) | 29,410 (16.4) |
|  | 70-74 years |  |  |  | 52,002 (17.3) | 15,368 (12.7) | 36,634 (20.4) |
|  | 65-69 years |  |  |  | 44,076 (14.6) | 12,216 (10.1) | 31,860 (17.7) |
|  | 60-64 years | 37,901 (23.0) | 9,520 (29.3) | 28,381 (21.5) |  |  |  |
|  | 55-59 years | 33,255 (20.2) | 7,298 (22.5) | 25,957 (19.6) |  |  |  |
|  | 50-54 years | 26,336 (16.0) | 5,268 (16.2) | 21,068 (15.9) |  |  |  |
|  | 45-49 years | 19,896 (12.1) | 3,548 (10.9) | 16,348 (12.4) |  |  |  |
|  | 40-44 years | 13,792 (8.4) | 2,224 (6.8) | 11,568 (8.8) |  |  |  |
|  | 35-39 years | 11,438 (6.9) | 1,770 (5.5) | 9,668 (7.3) |  |  |  |
|  | 30-34 years | 9,508 (5.8) | 1,339 (4.1) | 8,169 (6.2) |  |  |  |
|  | 18-29 years | 12,465 (7.6) | 1,508 (4.6) | 10,957 (8.3) |  |  |  |
| Area deprivation index quintile | |  |  |  |  |  |  |
|  | FDeP 5 (most deprived) | 40,570 (24.6) | 8,667 (26.7) | 31,903 (24.1) | 73,910 (24.5) | 29,063 (23.9) | 44,847 (24.9) |
|  | FDeP 4 | 30,736 (18.7) | 6,735 (20.7) | 24,001 (18.2) | 62,141 (20.6) | 25,368 (20.9) | 36,773 (20.5) |
|  | FDeP 3 | 29,454 (17.9) | 5,939 (18.3) | 23,515 (17.8) | 55,936 (18.6) | 21,964 (18.1) | 33,972 (18.9) |
|  | FDep 2 | 31,724 (19.3) | 5,762 (17.7) | 25,962 (19.7) | 52,529 (17.4) | 21,165 (17.4) | 31,364 (17.4) |
|  | FDep 1 (least deprived) | 32,107 (19.5) | 5,372 (16.5) | 26,735 (20.2) | 56,643 (18.8) | 23,835 (19.6) | 32,808 (18.3) |
| Residency in metropolitan France | |  |  |  |  |  |  |
|  | North-East region | 39,877 (24.2) | 9,580 (29.5) | 30,297 (22.9) | 82,035 (27.2) | 34,274 (28.2) | 47,761 (26.6) |
|  | North-West region | 24,361 (14.8) | 5,977 (18.4) | 18,384 (13.9) | 49,970 (16.6) | 20,708 (17.1) | 29,262 (16.3) |
|  | South-East region | 41,145 (25.0) | 7,578 (23.3) | 33,567 (25.4) | 85,278 (28.3) | 32,829 (27.0) | 52,449 (29.2) |
|  | South-West region | 11,769 (7.2) | 2,584 (8.0) | 9,185 (7.0) | 22,436 (7.4) | 9,542 (7.9) | 12,894 (7.2) |
|  | Ile-de-France region | 47,439 (28.8) | 6,756 (20.8) | 40,683 (30.8) | 61,440 (20.4) | 24,042 (19.8) | 37,398 (20.8) |
| Any pre-existing mental disorder | | 32,475 (19.7) | 32,475 (100.0) |  | 121,395 (40.3) | 121,395 (100.0) |  |
|  | Dementia | 1,509 (0.9) | 1,509 (4.6) |  | 66,030 (21.9) | 66,030 (54.4) |  |
|  | Depression | 8,681 (5.3) | 8,681 (26.7) |  | 40,739 (13.5) | 40,739 (33.6) |  |
|  | Anxiety disorders | 8,028 (4.9) | 8,028 (24.7) |  | 38,011 (12.6) | 38,011 (31.3) |  |
|  | Schizophrenia | 4,951 (3.0) | 4,951 (15.2) |  | 8,449 (2.8) | 8,449 (7.0) |  |
|  | Alcohol use disorders | 13,752 (8.4) | 13,752 (42.3) |  | 22,757 (7.6) | 22,757 (18.7) |  |
|  | Opioid use disorders | 1,939 (1.2) | 1,939 (6.0) |  | 1,149 (0.4) | 1,149 (0.9) |  |
|  | Down syndrome | 1,033 (0.6) | 1,033 (3.2) |  | 89 (0.0) | 89 (0.1) |  |
|  | Other learning disability | 3,108 (1.9) | 3,108 (9.6) |  | 1,969 (0.7) | 1,969 (1.6) |  |
|  | Other disorders with psychiatric ward admission | 633 (0.4) | 633 (1.9) |  | 2,236 (0.7) | 2,236 (1.8) |  |
| Any risk factor for severe COVID-19 | | 91,925 (55.9) | 23,754 (73.1) | 68,171 (51.6) | 245,387 (81.5) | 104,330 (85.9) | 141,057 (78.5) |
|  | Tobacco smoking | 24,084 (14.6) | 12,289 (37.8) | 11,795 (8.9) | 33,125 (11.0) | 17,302 (14.3) | 15,823 (8.8) |
|  | Obesity (BMI≥30 kg/m^2^) | 46,286 (28.1) | 10,548 (32.5) | 35,738 (27.1) | 71,099 (23.6) | 29,581 (24.4) | 41,518 (23.1) |
|  | Hypertension | 49,328 (30.0) | 13,294 (40.9) | 36,034 (27.3) | 221,490 (73.5) | 96,082 (79.1) | 125,408 (69.8) |
|  | Diabetes mellitus | 34,728 (21.1) | 8,495 (26.2) | 26,233 (19.9) | 100,434 (33.3) | 40,244 (33.2) | 60,190 (33.5) |
| Any severe somatic comorbidity | | 57,168 (34.7) | 20,013 (61.6) | 37,155 (28.1) | 208,744 (69.3) | 94,595 (77.9) | 114,149 (63.5) |
|  | Congestive heart failure | 11,664 (7.1) | 4,801 (14.8) | 6,863 (5.2) | 102,755 (34.1) | 49,526 (40.8) | 53,229 (29.6) |
|  | Peripheral vascular disease | 7,011 (4.3) | 3,216 (9.9) | 3,795 (2.9) | 47,745 (15.9) | 23,599 (19.4) | 24,146 (13.4) |
|  | Cerebrovascular disease | 7,698 (4.7) | 3,683 (11.3) | 4,015 (3.0) | 57,567 (19.1) | 33,416 (27.5) | 24,151 (13.4) |
|  | Chronic pulmonary disease | 20,414 (12.4) | 7,649 (23.6) | 12,765 (9.7) | 61,557 (20.4) | 28,368 (23.4) | 33,189 (18.5) |
|  | Hemiplegia | 7,645 (4.6) | 3,761 (11.6) | 3,884 (2.9) | 26,544 (8.8) | 14,755 (12.2) | 11,789 (6.6) |
|  | Moderate or severe liver disease | 3,059 (1.9) | 2,248 (6.9) | 811 (0.6) | 4,768 (1.6) | 3,434 (2.8) | 1,334 (0.7) |
|  | Mild liver disease | 7,994 (4.9) | 3,786 (11.7) | 4,208 (3.2) | 11,083 (3.7) | 6,311 (5.2) | 4,772 (2.7) |
|  | Moderate or severe renal disease | 7,727 (4.7) | 2,771 (8.5) | 4,956 (3.8) | 61,667 (20.5) | 31,318 (25.8) | 30,349 (16.9) |
|  | Metastatic solid tumour | 6,975 (4.2) | 2,558 (7.9) | 4,417 (3.3) | 19,033 (6.3) | 7,293 (6.0) | 11,740 (6.5) |
|  | Solid tumour without metastasis | 6,653 (4.0) | 2,440 (7.5) | 4,213 (3.2) | 36,412 (12.1) | 15,995 (13.2) | 20,417 (11.4) |
|  | Haematological malignancy | 3,423 (2.1) | 939 (2.9) | 2,484 (1.9) | 11,803 (3.9) | 4,349 (3.6) | 7,454 (4.1) |
|  | AIDS | 1,298 (0.8) | 418 (1.3) | 880 (0.7) | 504 (0.2) | 190 (0.2) | 314 (0.2) |
|  | Transplant recipient | 4,161 (2.5) | 1,327 (4.1) | 2,834 (2.1) | 4,236 (1.4) | 1,753 (1.4) | 2,483 (1.4) |
| Delay between the latest acute hospital discharge and first COVID-19 record | |  |  |  |  |  |  |
|  | SARS-CoV-2 infection during hospital care | 15,308 (9.3) | 6,702 (20.6) | 8,606 (6.5) | 55,119 (18.3) | 28,684 (23.6) | 26,435 (14.7) |
|  | Previous discharge in the last 3 months | 21,570 (13.1) | 7,768 (23.9) | 13,802 (10.4) | 61,430 (20.4) | 27,817 (22.9) | 33,613 (18.7) |
|  | Previous discharge in the last 4-12 months | 29,190 (17.7) | 6,004 (18.5) | 23,186 (17.5) | 63,266 (21.0) | 24,817 (20.4) | 38,449 (21.4) |
|  | Previous discharge in the last 2-3 years | 20,201 (12.3) | 5,857 (18.0) | 14,344 (10.9) | 51,708 (17.2) | 23,635 (19.5) | 28,073 (15.6) |
|  | Previous discharge in the last 4-9 years | 29,894 (18.2) | 4,041 (12.4) | 25,853 (19.6) | 42,688 (14.2) | 12,577 (10.4) | 30,111 (16.8) |
|  | No previous admission in the last 9 years | 48,428 (29.4) | 2,103 (6.5) | 46,325 (35.1) | 26,948 (8.9) | 3,865 (3.2) | 23,083 (12.8) |
| Outcomes | |  |  |  |  |  |  |
|  | Death over the follow-up | 10,881 (6.6) | 4,475 (13.8) | 6,406 (4.8) | 93,009 (30.9) | 42,508 (35.0) | 50,501 (28.1) |
|  | Salvage therapy including: | 40,897 (24.8) | 8,588 (26.4) | 32,309 (24.5) | 52,089 (17.3) | 12,912 (10.6) | 39,177 (21.8) |
|  | Intensive care unit (ICU) admission | 37,211 (22.6) | 7,883 (24.3) | 29,328 (22.2) | 44,475 (14.8) | 10,507 (8.7) | 33,968 (18.9) |
|  | 1) Extracorporeal membrane oxygenation | 1,030 (0.6) | 114 (0.4) | 916 (0.7) | 257 (0.1) | 25 (0.0) | 232 (0.1) |
|  | 2) Invasive mechanical respiratory support | 22,337 (13.6) | 5,258 (16.2) | 17,079 (12.9) | 31,428 (10.4) | 7,359 (6.1) | 24,069 (13.4) |
|  | 3) Continuous positive airway pressure | 11,269 (6.8) | 1,673 (5.2) | 9,596 (7.3) | 13,343 (4.4) | 3,195 (2.6) | 10,148 (5.6) |

# Table G. 120-day mortality and salvage therapy risks by category of pre-existing mental disorders (n=465 750)

| **Category of pre-existing mental disorder** | **Patients** | | **Male, %** | | **Age, median (IQR) years** | | **≥ 2 mental disorders, %** | | **120-day mortality** | | | | | | | **Salvage therapy** | | | | | | |  |
| --- | --- | --- | --- | --- | --- | --- | --- | --- | --- | --- | --- | --- | --- | --- | --- | --- | --- | --- | --- | --- | --- | --- | --- |
|  |  |  |  |  |  |  |  |  | **Risk predicted, %**  **(95% CI)** | | **Risk observed, %**  **(95% CI)** | | **Excess risk, %**  **(95% CI)** | | **Excess risk, No patients (95% CI)** | **Risk predicted, %**  **(95% CI)** | | **Risk observed, %**  **(95% CI)** | | | **Excess risk, %**  **(95% CI)** | **Excess risk, No patients**  **(95% CI)** |  |
| No mental disorder | 311 880 | 56.4 | | 68  (55;80) | | 0 | | 13.3  (13.2;13.4) | | 18.2  (18.0;18.5) | | 5.0  (4.7;5.2) | | 15 512.7  (14 678.7;16 346.7) | | | 18.8  (18.7;18.9) | | 22.9  (22.6;23.2) | 4.2  (3.8;4.5) | | 12 965.8  (11 993.8;13 937.7) | |
| Any pre-existing mental disorder | 153 870 | 49.0 | | 80  (67;88) | | 35.4 | | 21.2  (21.0;21.4) | | 30.5  (30.0;31.0) | | 9.3  (8.9;9.8) | | 14 343.1  (13 621.9;15 064.4) | | | 18.0  (17.9;18.2) | | 14.0  (13.6;14.4) | -4.1  (-4.4;-3.7) | | -6 242.4  (-6 805.4;-5 679.4) | |
| Dementia | 67 539 | 43.8 | | 86  (80;90) | | 44.5 | | 24.5  (24.2;24.7) | | 35.6  (34.8;36.3) | | 11.1  (10.4;11.9) | | 7 512.3  (7 003.1;8 021.5) | | | 13.2  (13.0;13.4) | | 5.7  (5.3;6.1) | -7.5  (-7.9;-7.1) | | -5 065.0  (-5 326.3;-4 803.7) | |
| Depression | 49 420 | 33.1 | | 81  (70;88) | | 74.2 | | 21.8  (21.5;22.2) | | 30.9  (30.0;31.8) | | 9.1  (8.2;9.9) | | 4 474.9  (4 069.0;4 880.8) | | | 16.9  (16.6;17.1) | | 12.4  (11.7;13.0) | -4.5  (-5.1;-3.9) | | -2 223.3  (-2 526.5;-1 920.1) | |
| Anxiety disorders | 46 039 | 33.1 | | 82  (70;89) | | 76.4 | | 23.0  (22.6;23.3) | | 31.7  (30.8;32.6) | | 8.8  (7.9;9.6) | | 4 032.4  (3 635.8;4 429.0) | | | 16.2  (15.9;16.4) | | 11.6  (11.0;12.2) | -4.5  (-5.2;-3.9) | | -2 093.7  (-2 380.3;-1 807.2) | |
| Schizophrenia | 13 400 | 47.8 | | 70  (59;81) | | 75.0 | | 15.0  (14.5;15.4) | | 23.5  (22.0;25.0) | | 8.5  (7.1;10.0) | | 1 145.5  (949.3;1 341.8) | | | 21.5  (20.9;22.0) | | 18.0  (16.5;19.4) | -3.5  (-4.9;-2.1) | | -466.1  (-653.3;-278.9) | |
| Alcohol use disorders | 36 509 | 77.7 | | 69  (59;77) | | 25.9 | | 19.0  (18.6;19.4) | | 26.6  (25.6;27.5) | | 7.5  (6.6;8.5) | | 2 753.2  (2 416.1;3 090.3) | | | 26.7  (26.4;27.1) | | 23.8  (22.8;24.7) | -2.9  (-3.9;-2.0) | | -1 069.1  (-1 413.9;-724.4) | |
| Opioid use disorders | 3 088 | 56.3 | | 57  (46;74) | | 57.6 | | 15.1  (13.8;16.3) | | 17.7  (14.8;20.7) | | 2.6  (-0.1;5.4) | | 81.7  (-2.7;166.1) | | | 22.9  (21.8;24.1) | | 22.6  (19.3;25.9) | -0.3  (-3.6;2.9) | | -10.5  (-111.3;90.3) | |
| Down syndrome | 1 122 | 57.4 | | 52  (41;58) | | 16.6 | | 4.8  (4.3;5.3) | | 19.6  (14.8;24.5) | | 14.8  (10.0;19.6) | | 165.9  (112.4;219.5) | | | 17.4  (16.4;18.4) | | 21.6  (16.5;26.6) | 4.2  (-0.8;9.2) | | 46.8  (-9.2;102.9) | |
| Other learning disabilities | 5 077 | 56.3 | | 61  (51;69) | | 46.3 | | 9.1  (8.6;9.7) | | 18.7  (16.4;20.9) | | 9.5  (7.4;11.7) | | 484.4  (374.3;594.4) | | | 20.4  (19.7;21.1) | | 18.0  (15.8;20.3) | -2.4  (-4.6;-0.1) | | -120.2  (-234.4;-6.0) | |
| Other disorder with psychiatric ward admission | 2 869 | 55.0 | | 80  (67;88) | | 0 | | 21.7  (20.3;23.0) | | 33.4  (29.7;37.1) | | 11.7  (8.3;15.2) | | 336.9  (237.2;436.5) | | | 16.8  (15.8;17.8) | | 15.3  (12.4;18.2) | -1.5  (-4.3;1.3) | | -42.5  (-123.0;38.0) | |

# Fig A. Associations of pandemic periods and pre-existing mental disorders with 120-day mortality risk among inpatients with symptomatic COVID-19 aged 18-64 years (n=164 591)


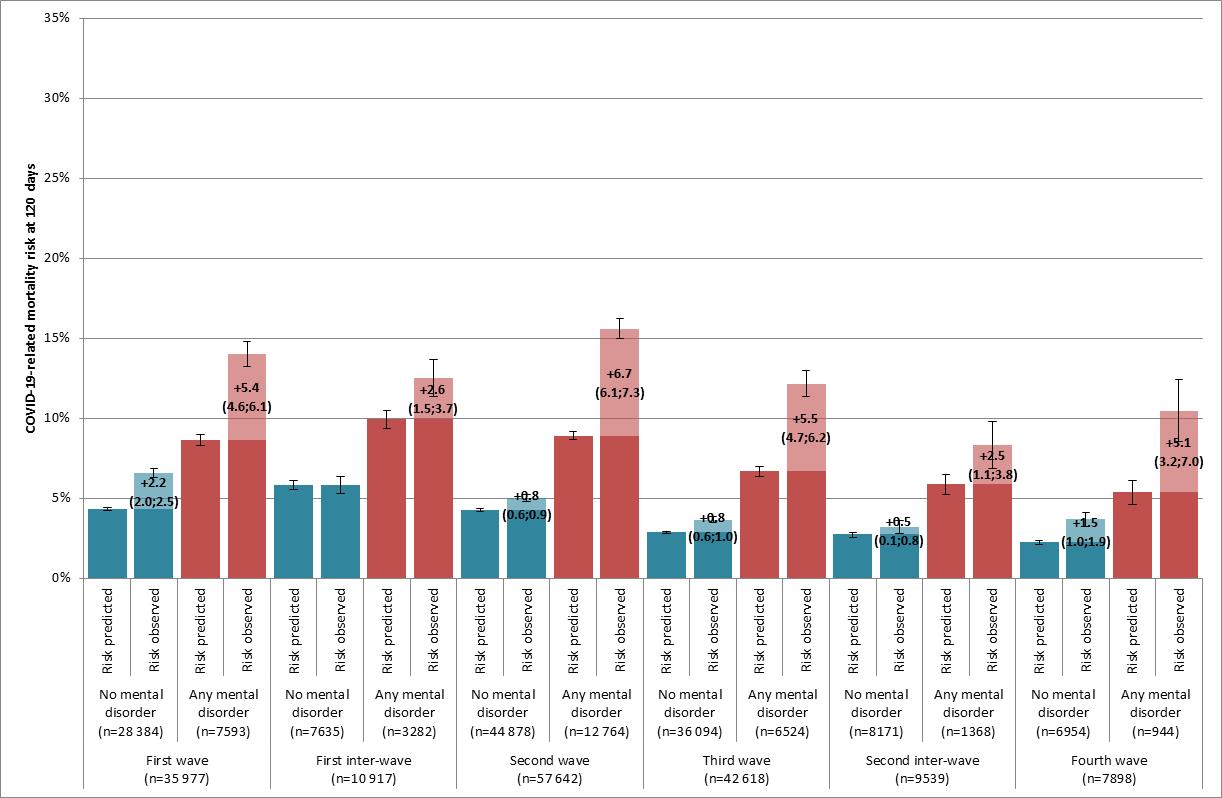


Patients with (without) mental disorders: 4475 (6406) deaths; risk predicted: 8.3% [7.9-8.6] (3.8% [3.7-3.9]); excess risk: +5.5% [4.7-6.3] (+1.1 [0.8-1.3])

# Fig B. Associations of pandemic periods and pre-existing mental disorders with 120-day mortality risk among inpatients with symptomatic COVID-19 aged 65 years and above (n=301 159)


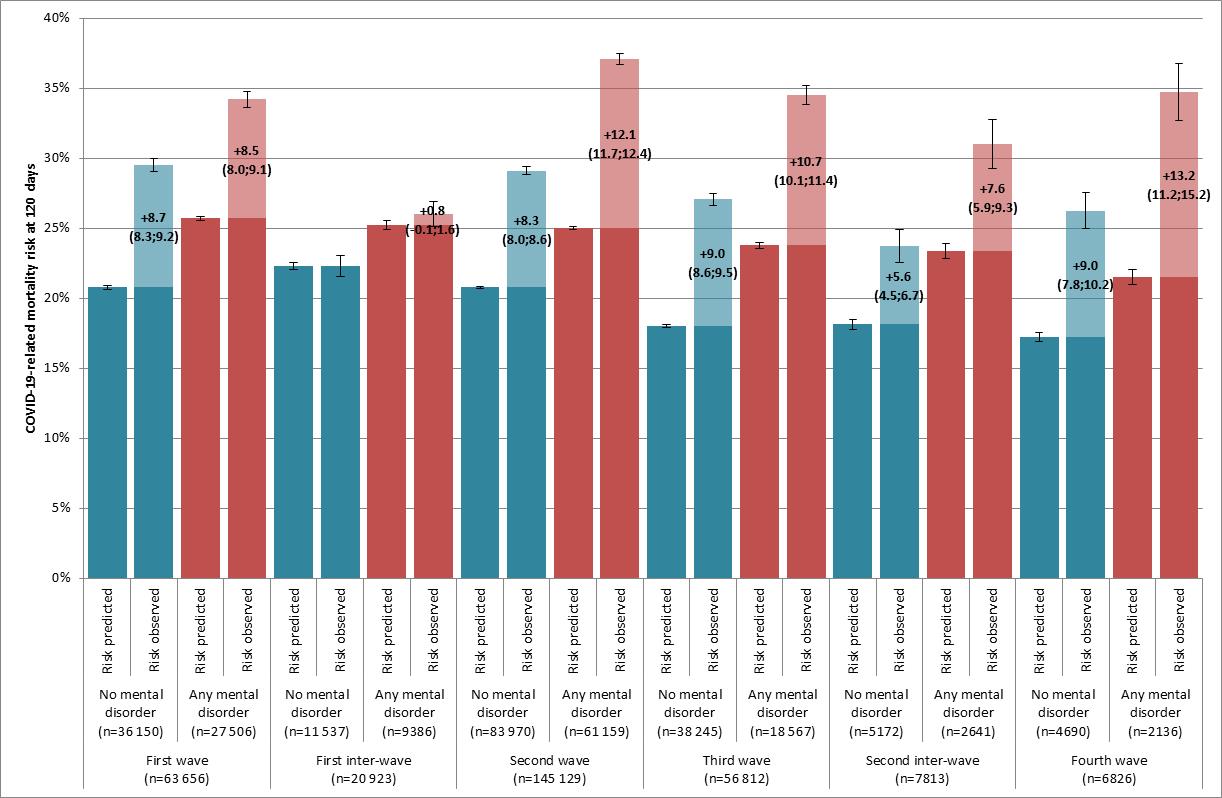


Patients with (without) mental disorders: 42 508 (50 501) deaths; risk predicted: 24.9% [24.7-25.1] (20.1% [20.0-20.3]); excess risk: +10.1% [9.6-10.7] (+8.0% [7.5-8.4])

# Fig C. Associations of pandemic periods and pre-existing mental disorders with salvage therapy rate among inpatients with symptomatic COVID-19 aged 18-64 years (n=164 591)


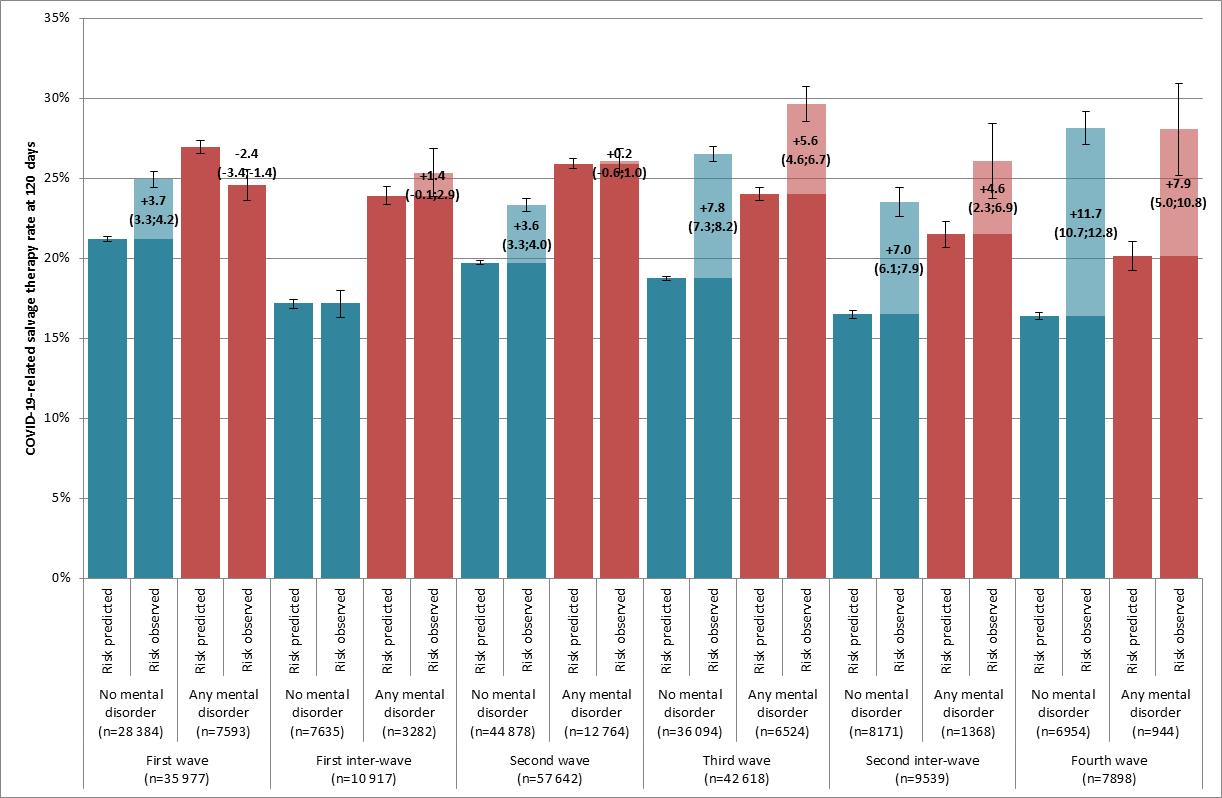


Patients with (without) mental disorders: 8588 (32 309) admissions; risk predicted: 25.2% [24.8-25.6] (19.2% [19.0-19.4]); excess risk: +1.2% [0.1-2.3] (+5.2% [4.7-5.7])

# Fig D. Associations of pandemic periods and pre-existing mental disorders with salvage therapy rate among inpatients with symptomatic COVID-19 aged 65 years and above (n=301 159)


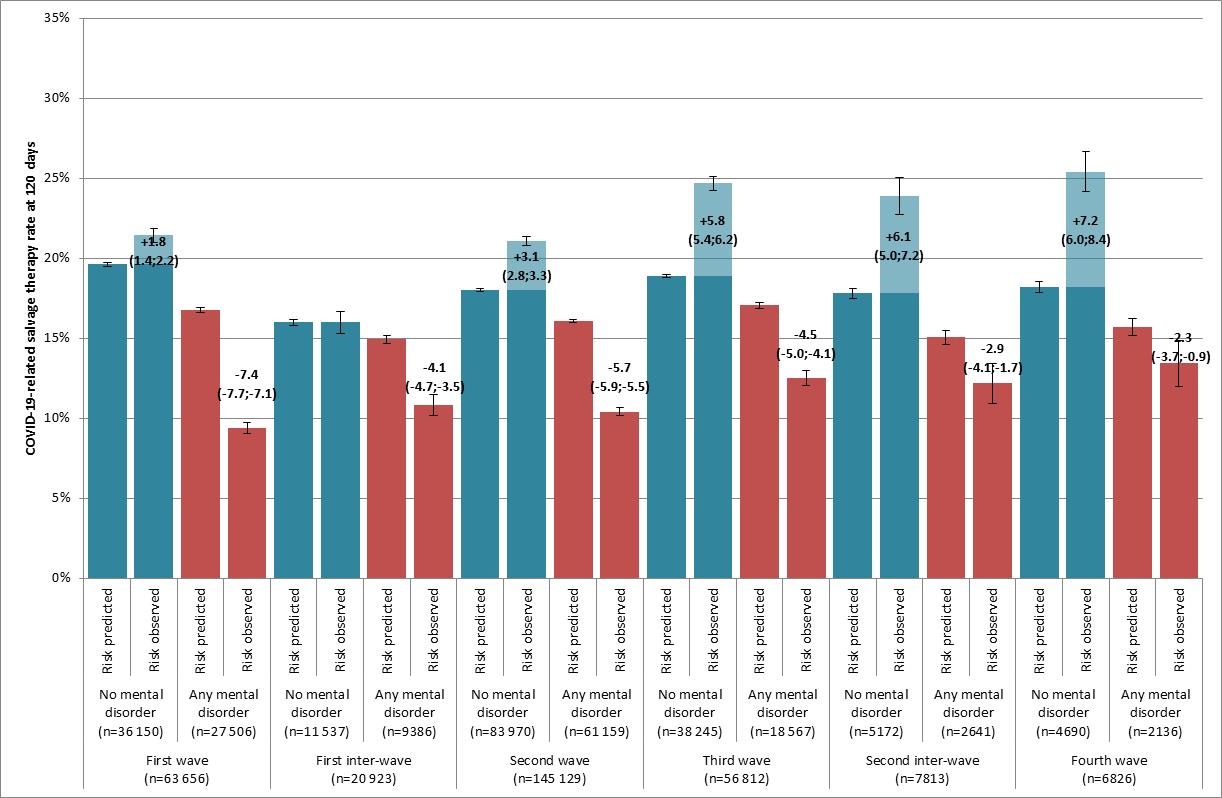


Patients with (without) mental disorders: 1 2912 (39 177) admissions; risk predicted: 16.3% [16.1-16.4] (18.4% [18.3-18.5]); excess risk: -5.7% [-6.0;-5.3] (+3.4% [3.0-3.8])

# Fig E. Associations of pandemic periods and pre-existing mental disorders with 120-day mortality risk among inpatients with COVID-19-related respiratory symptoms (n=371 016)


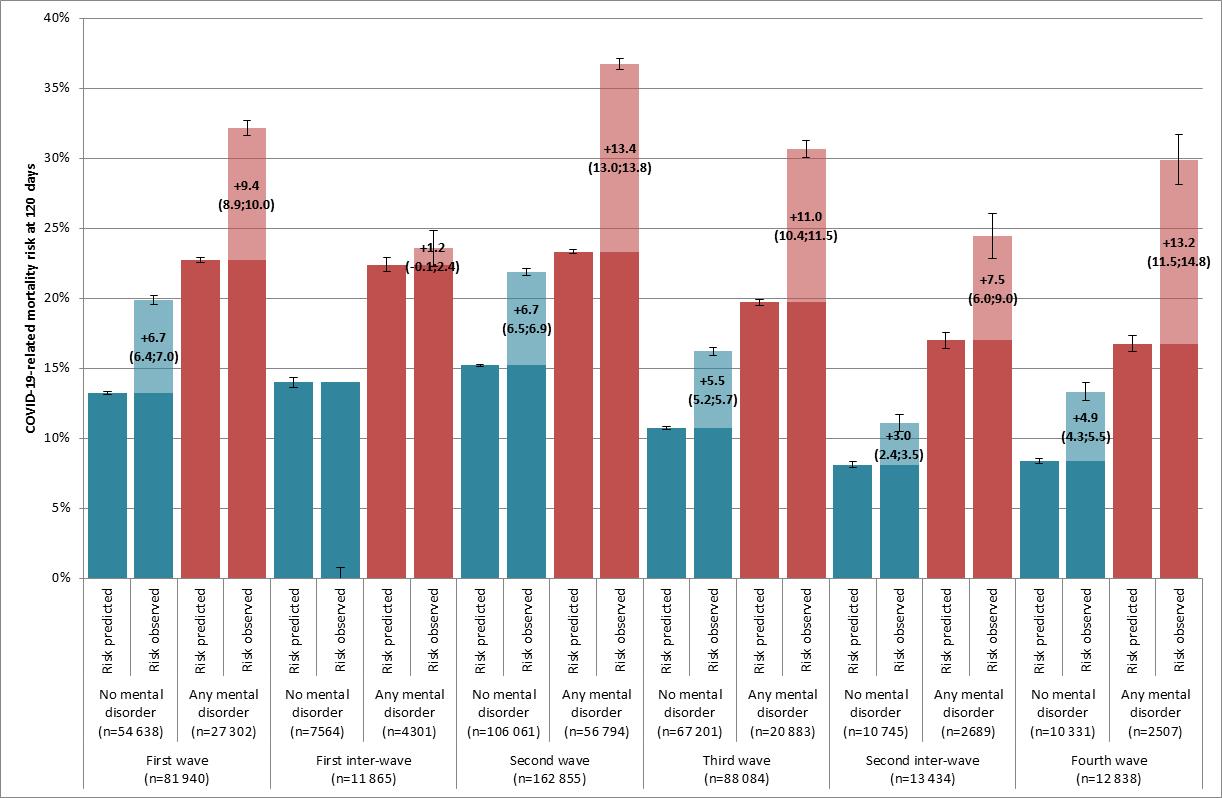


Patients with (without) mental disorders: 38 483 (48 631) deaths; risk predicted: 22.2% [22.0-22.4] (13.0% [12.9-13.1]); excess risk: +11.4% [10.9-12.0] (+6.0% [5.7-6.2])

# Fig F. Associations of pandemic periods and pre-existing mental disorders on salvage therapy rate among inpatients with COVID-19-related respiratory symptoms (n=371 016)


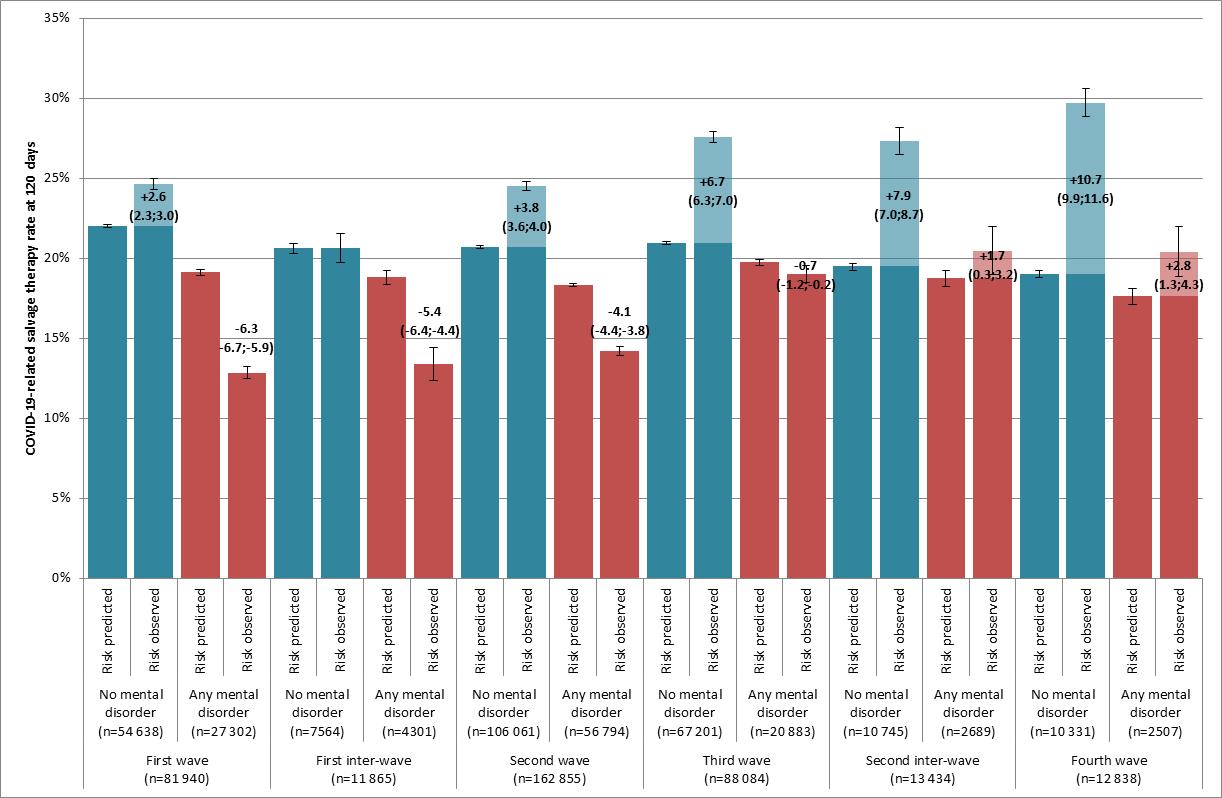


Patients with (without) mental disorders: 17 201 (65 591) admissions; risk predicted: 18.8% [18.6-19.0] (20.9% [20.8-21.0]); excess risk: -3.8% [-4.2;-3.3] (+4.6 [4.3-5.0])

# Fig G. Associations of pandemic periods and pre-existing mental disorders with 120-day mortality risk among inpatients admitted for symptomatic COVID-19 (n= 395 323)


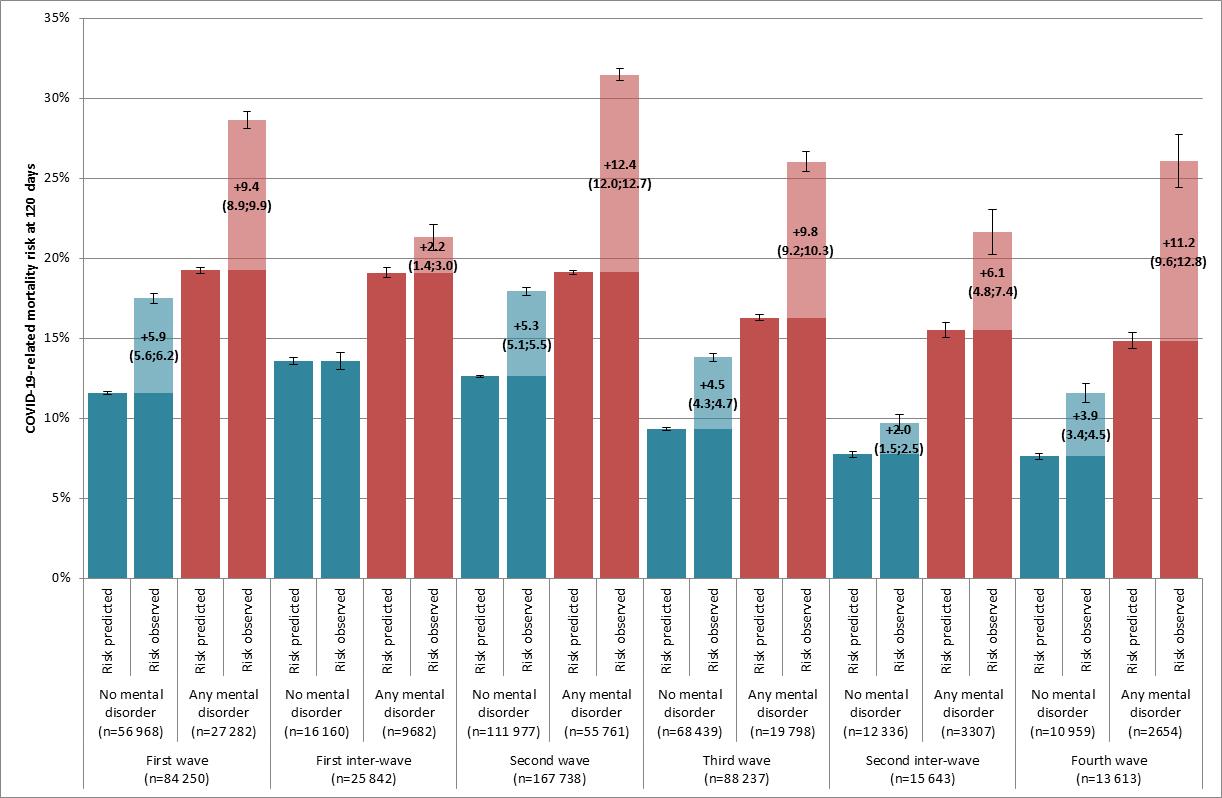


Patients with (without) mental disorders: 33 994 (44 182) deaths; risk predicted: 18.5% [18.3-18.7] (11.2% [11.1-11.3]); excess risk: +10.2% [9.7-10.7] ( +4.7 [4.5-5.0])

# Fig H. Associations of pandemic periods and pre-existing mental disorders on salvage therapy rate among inpatients admitted for symptomatic COVID-19 (n= 395 323)


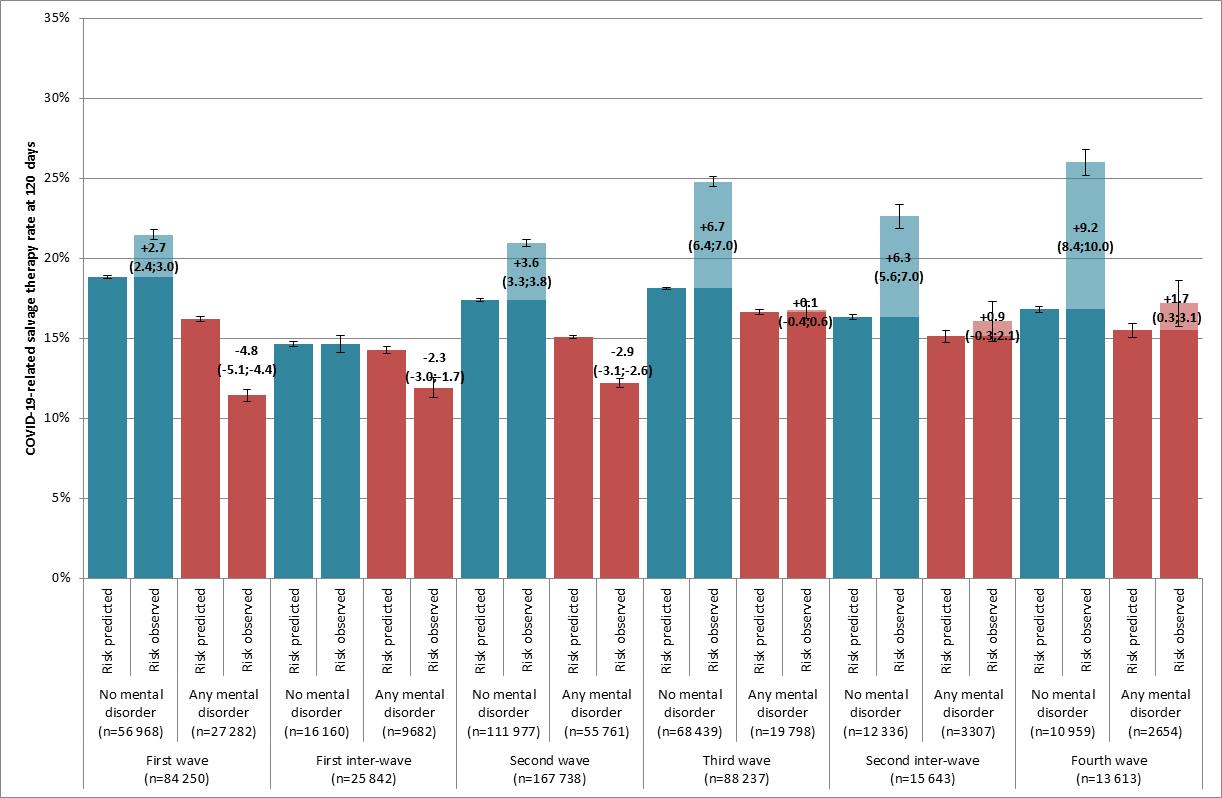


Patients with (without) mental disorders: 15 392 (60 700) admissions; risk predicted: 15.5% [15.4-15.7] (17.6% [17.5-17.7]); excess risk: -2.6 [-3.0;-2.1] (+4.3 [4.0-4.6])

# Fig I. Associations of pandemic periods and pre-existing mental disorders with intensive-care unit admission rate among inpatients with symptomatic COVID-19 (n=465 750)


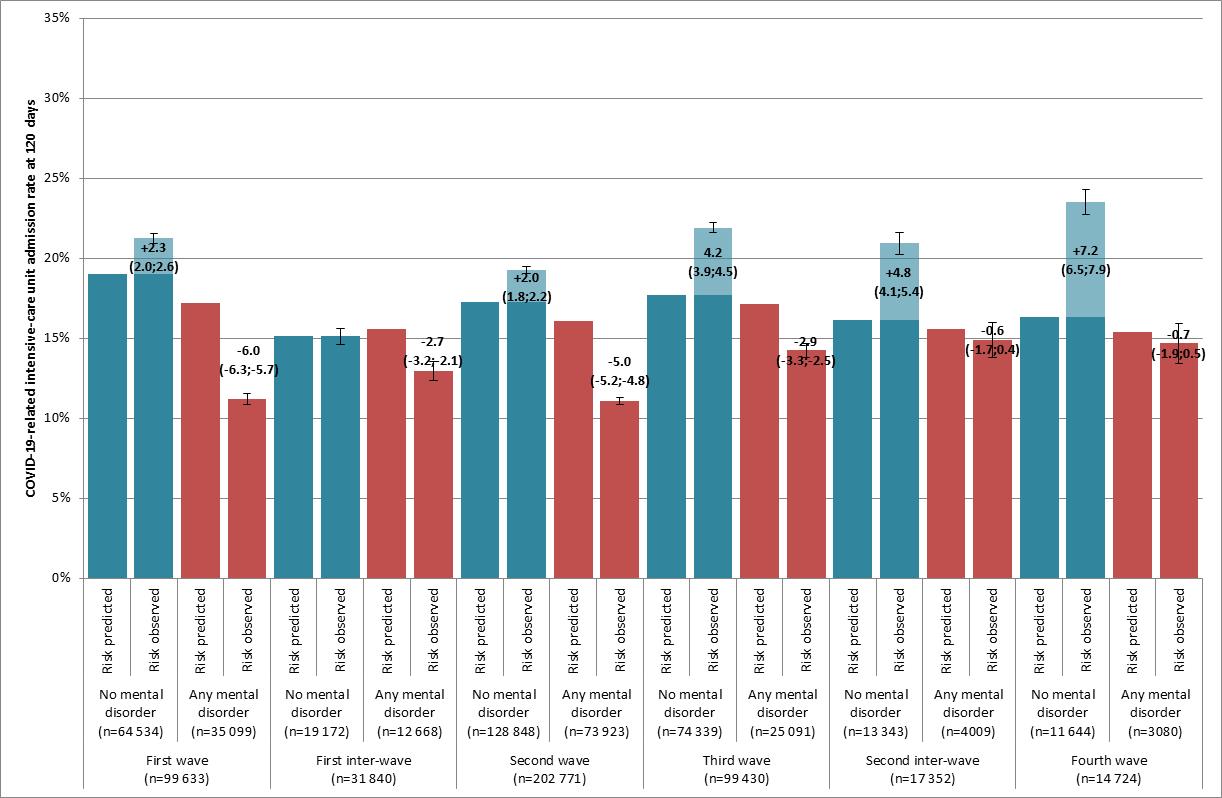


Patients with (without) mental disorders: 18 390 (63 296) admissions; risk predicted: 16.5% [16.3-16.6] (17.5% [17.4-17.6]); excess risk: -4.5% [-4.9;-4.2] (+2.8 [2.5-3.1])

# Fig J. Associations of pandemic periods and pre-existing mental disorders with 28-day mortality risk among inpatients with symptomatic COVID-19 (n=465 750)


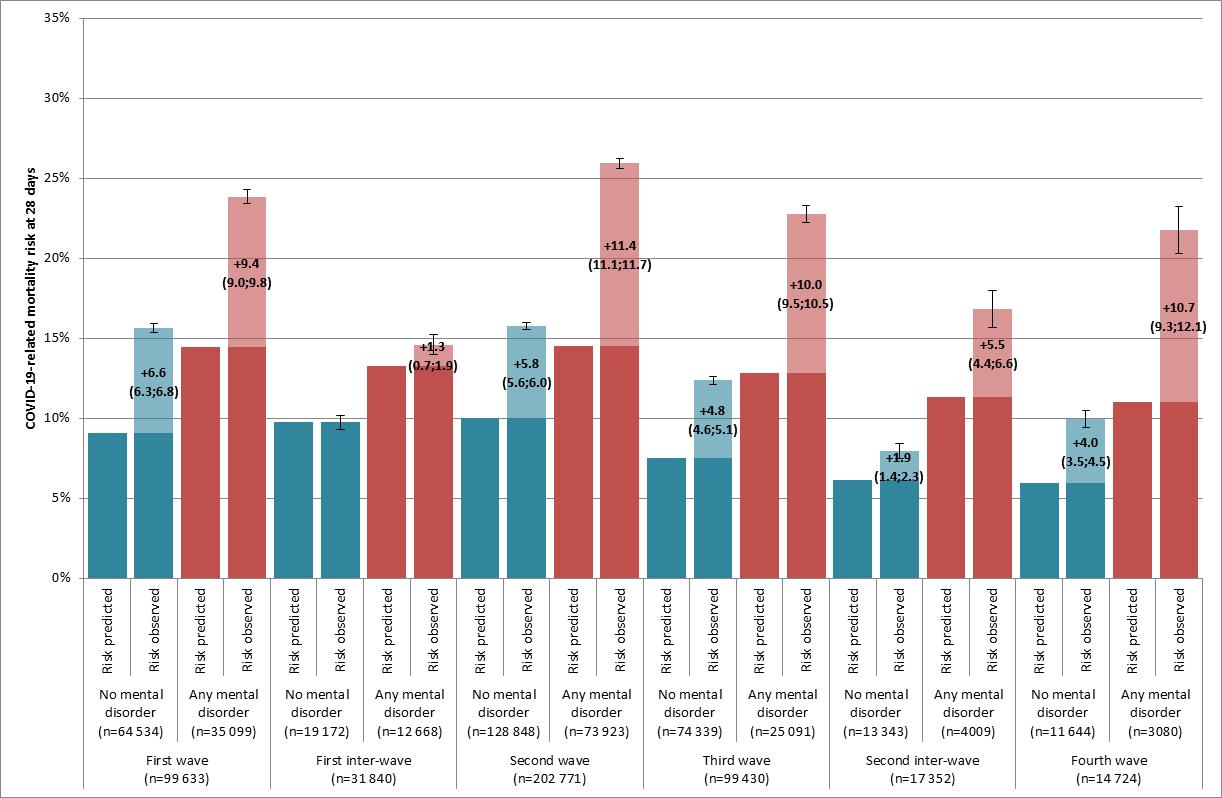


Patients with (without) mental disorders: 36 465 (43 709) deaths; risk predicted: 14.0% [13.9-14.1] (8.9% [8.8-8.9]): excess risk: +9.7% [9.3-10.2] (+5.1% [4.9-5.4])

# Fig K. Associations of pandemic periods and pre-existing mental disorders with mortality risk at first acute hospital discharge among inpatients with symptomatic COVID-19 (n=465 750)


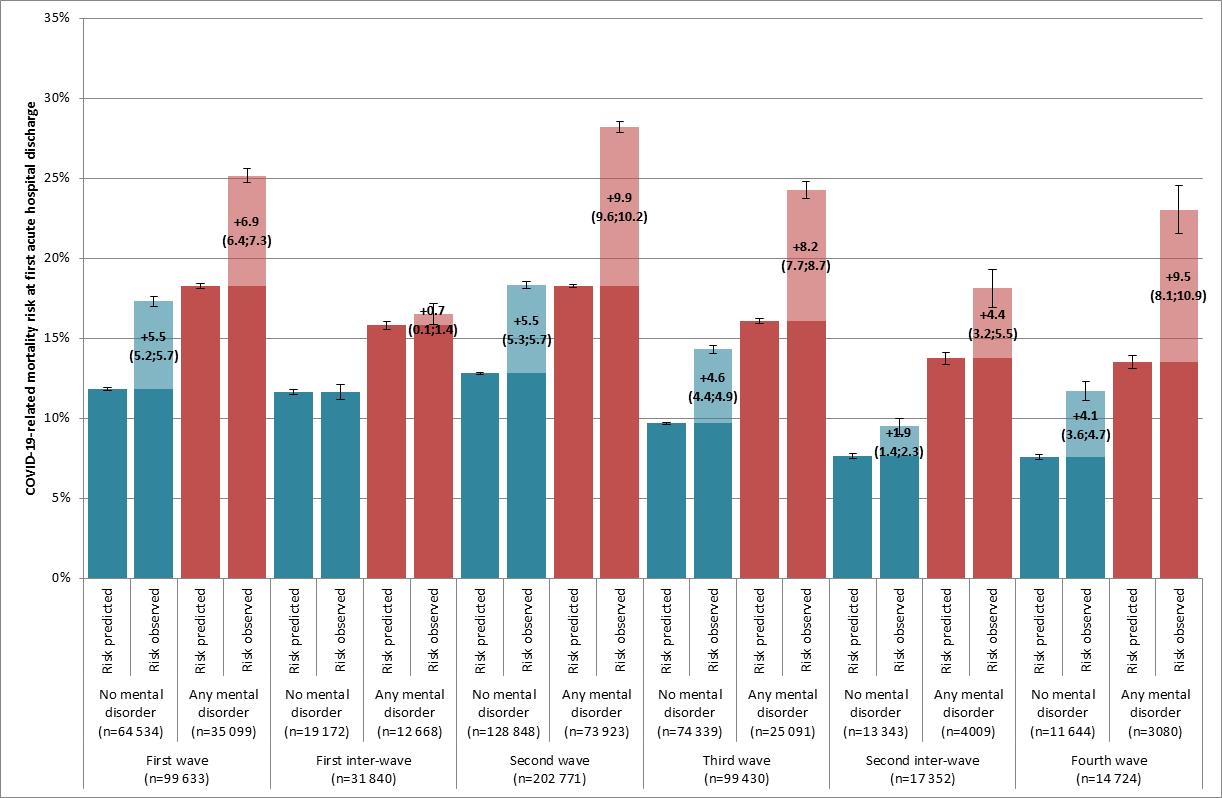


Patients with (without) mental disorders: 39 310 (50 310) deaths; risk predicted: 17.5% [17.4-17.6] (11.4% [11.3-11.5]): excess risk: +8.0% [7.6-8.5] (+4.8 [4.5-5.0])
