# Supplementary material for: Mental disorders, COVID-19-related life-saving measures and mortality in France: A nationwide cohort study
Source: PLoS Med. 2023 Feb 6;20(2):e1004134. doi: 10.1371/journal.pmed.1004134 (PMC10089350; doi:10.1371/journal.pmed.1004134)
Supplement: Attachment — Submitted filename: plosm.docx [file pmed.1004134.s002.docx]

**Title: Mental disorders and COVID-19-related life-saving measures and mortality: a nationwide, retrospective hospital cohort study over an 18-month period in France**

The manuscript has great public health value and well presented. Just I have the following few comments.

1. Why authors used categorical data instead of continuous scores?
2. On page 7 and 8, the authors stated that *“Our case study of salvage therapy suggests that triage decisions for life-saving measures at hospital were 259 disproportionately taken to maximize health benefits at the expense of COVID-19 patients with mental disorders: salvage therapy rates due to caseload surges were significantly higher than expected in 261 patients without mental disorders (+4.2% [95% CI, 3.8-4.5]) and lower in patients with mental disorders 262 (-4.1% [95% CI, -4.4;-3.7]”.* What can be the possible explanations for this finding? can stigma against people with mental illness also be one possible explanation?
3. Title of the tables and graphs should give full information.
