# Supplementary material for: Mental disorders, COVID-19-related life-saving measures and mortality in France: A nationwide cohort study
Source: PLoS Med. 2023 Feb 6;20(2):e1004134. doi: 10.1371/journal.pmed.1004134 (PMC10089350; doi:10.1371/journal.pmed.1004134)
Supplement: Attachment — Submitted filename: PMEDICINE-D-22-02340R1 Point-by-point responses.docx [file pmed.1004134.s003.docx]

# Point-by-point responses PMEDICINE-D-22-02340R1

## Comment from the Academic Editor

I do not see analyses which attempt to draw a causal inference between access to salvage therapy (which would have occurred during hospitalization and, always, before death) and mortality. This, in my view, diminishes the potential impact of the analyses. That said, the data is all there and I am sure a statistician with the relevant expertise (for e.g. in path/mediation analysis) could carry out analysis to examine the proportion of the excess mortality risk which could be attributed to lower access to salvage therapy. This, to my mind, would make this paper stand out from all others I know on the excess mortality risk for persons with mental disorders.

**We thank the academic editor for this suggestion. We previously conducted a simultaneous probit multivariate model with three dependent variables (access to salvage therapy, mortality with salvage therapy, and mortality without salvage therapy) showing that independent associations of multiple categories of mental disorders with mortality risk (except for opioid use disorders) were usually found also in patients with or without salvage therapy (Table S3).**

**Following the academic editor’s suggestion, we conducted a series of causal mediation analyses to assess the direct effect of each category of mental disorder on all-cause mortality that is not due to mediation or interaction (so-called “controlled direct effect” in the counterfactual framework). We found similarly that, except for opioid use disorders, each category of mental disorders (except for opioid use disorders) had a direct effect on higher mortality risk in patients without salvage therapy (all adjusted OR>1 and p<0.001).**

**However, both analyses pointed to unobserved confounding factors associated with a higher access to salvage therapy in patients with better prognosis, all other observed factors being equal. Such unobserved heterogeneity was directly assessed in the simultaneous probit multivariate model (significant rho correlation coefficients between equations), with potential impact in causal mediation analyses that rely on the assumption of “no unobserved confounding factors” (Valeri et al. Psychol Methods 2013). Accordingly, we found also that two categories of mental disorder (dementia, anxiety disorders) had unexpectedly a controlled direct effect on lower mortality risk in patients with salvage therapy (adjusted OR<1 and p<0.001).**

**Following the editor’s suggestion and previous considerations, we have cautiously added previous and new analyses as sensitivity analyses in the revised manuscript (Methods page 6, line 213 of the “marked-up” revision; Results page 8, line 275; Appendix S4 Table).**

**References**

Valeri, L. and T. J. Vanderweele (2013). "Mediation analysis allowing for exposure-mediator interactions and causal interpretation: theoretical assumptions and implementation with SAS and SPSS macros." Psychol Methods **18**(2): 137-150.

## Requests from the editors

Please revise your title to ‘Mental disorders, COVID-19-related life-saving measures and mortality in France: a nationwide cohort study’, or similar.

**The title has been changed as suggested.**

Line 31: ‘have shown’ rather than ‘showed’.

**This has been corrected.**

Abstract Background: The final sentence should clearly state the study question.

**We have stated more clearly the study question by adding: “Our study objectives were to assess independent associations between various categories of mental disorders and COVID-19-related mortality in a nationwide sample of COVID-19 inpatients discharged over 18 months and the potential role of salvage therapy triage to explain these associations.”**

Abstract Methods and Findings:

* Please include the years during which the study took place.

**We have detailed the study period: “between February 24, 2020, and August 28, 2021”**

* In the last sentence of the Abstract Methods and Findings section, please describe the main limitation(s) of the study's methodology.

**We have added the main limitations of our study: “The main limitations of our study point to the assessment of COVID-19-related mortality at 120 days and potential coding bias of medical information recorded in hospital claims data, although the main study findings were consistently reproduced in multiple sensitivity analyses.”**

Line 53: ‘odds of’ rather than ‘odds in’.

**This has been corrected.**

Please ensure you address reviewer comments pertaining to the assessment of mental disorders (e.g., reviewer #3, comment #4) as this issue has the potential to introduce bias in the findings.

**Following the reviewer’s concern, we conducted another sensitivity analysis on the effects of the time window used to define covariates from the national hospital discharge database. We found reassuringly that the main study results were robust to a left-truncation of all discharge diagnosis data recorded before Jan 1, 2019, although the magnitude of the problem would be unduly underestimated by a lower prevalence rate of pre-existing mental disorders. Please, see below our detailed response to comment #4 of reviewer #3.**

Please provide the rationale behind your decision to categorise Down’s syndrome and other learning disabilities as mental disorders. As noted by reviewer #1, it may be inappropriate to group genetic disorders associated with both mental and physical disabilities alongside other mental disorders.

**We agree with the editors and reviewer #1 that including Down’s syndrome among mental disorders may be controversial, although they have in common stigmatization and higher risk of premature death (Harris et al. Br J Psychiatry 1998). We identified mental disorders based on previous meta-analyses on the associations of mental disorders with COVID-19-related mortality (Vai et al. Lancet Psychiatry 2021, Fond et al. JAMA Psychiatry 2021). Both meta-analyses included Down’s syndrome and mental retardation either explicitly as a search term (Vai et al. Lancet Psychiatry 2021) or implicitly by selecting “studies that included participants with a diagnosis of mental health disorders according to the DSM or any version of the International Classification of Diseases (ICD)" (Fond et al. JAMA Psychiatry 2021). Regarding ICD-10 used to code discharge diagnoses in France, its Chapter V “Mental and behavioral disorders“ (F00-F99) includes “Mental retardation” (F70-F79). While Down's syndrome (Q90) is classified in Chapter XVII “Congenital malformations, deformations and chromosomal abnormalities” (Q00-Q99), these patients are also often recorded with F7x codes to characterize the level of mental retardation. In our study, 222 (20.9%) of 1122 patients recorded with Q90 were also recorded with F7x codes.**

**We clarified in the revised manuscript that “we identified pre-existing mental disorders based on previous meta-analyses” (Methods/procedures page 6 line 190 of the “marked-up” revision).**

At this stage, we ask that you include a short, non-technical Author Summary of your research to make findings accessible to a wide audience that includes both scientists and non-scientists. The Author Summary should immediately follow the Abstract in your revised manuscript. This text is subject to editorial change and should be distinct from the scientific abstract. Please see our author guidelines for more information: https://journals.plos.org/plosmedicine/s/revising-your-manuscript#loc-author-summary

**We have added an Author Summary after the Abstract.**

Please place citations before punctuation.

**This has been corrected throughout the revised manuscript.**

Introduction: Please address past research and explain the need for and potential importance of your study. Indicate whether your study is novel and how you determined that. If there has been a systematic review of the evidence related to your study (or you have conducted one), please refer to and reference that review and indicate whether it supports the need for your study.

**We have expanded the Introduction to better address past research (Introduction page 4 lines 106-116 of the “marked-up” revision) and the novelty of our study (Introduction page 4 lines 130-136 of the “marked-up” revision).**

**Systematic reviews and meta-analyses were formerly cited (Vai et al. Lancet Psychiatry 2021, Fond et al. JAMA Psychiatry 2021) and used to describe past research.**

Please define salvage therapy in your Introduction.

**We have added that salvage therapy relates to intensive-care unit admission or life-saving respiratory support in our study (Introduction page 4 line 135 of the “marked-up” revision).**

Please conclude the Introduction with a clear description of the study question or hypothesis.

**We have more clearly stated our study objectives and hypothesis (Introduction page 4 lines 121-136 of the “marked-up” revision).**

As noted by reviewer #3 (comment #3), the method used to attribute COVID-19 mortality requires justification as the editors felt the current approach could result in misclassification bias.

**In our opinion, “COVID-19-related mortality” is hard to define, especially with respect to time frames when a death should be attributed to COVID-19. In order to assess the potential impact of different time frames, we formerly conducted two sensitivity analyses using 28-day mortality (Figure S10) or in-hospital mortality at first discharge (Figure S11), and found that the main study results with 120-day mortality (Figure 2) were robust to a different timing of mortality assessment. In addition, we originally addressed this issue by disentangling the effects of patient vulnerability and COVID-19 caseload surges in hospitals on the outcomes of COVID-19 patients with pre-existing mental disorders and found that mortality risks predicted at baseline (in relation to higher vulnerability level) and excess mortality risks (due to COVID-19 caseload surges) were significantly higher in patients with pre-existing mental disorders compared to others (Figure 2). This phenomenon was consistently found in all sensitivity analyses.**

**We clarified this original set of analyses in the revised manuscript (Methods/Statistics analysis page 6 lines 218-235) and implications of the sensitivity analyses on the timing of mortality assessment (Discussion/limitations page 11 lines 388-392). Please, see also below our detailed response to comment #3 of reviewer #3.**

Line 102: As noted by reviewer #1, this statement requires clarification, as your study does rely on data from human participants.

**Data management and secondary analyses of the National Hospital Discharge database were performed in accordance with French laws for this type of research studies (“reference methodology MR-005”). Accordingly, the approval of an Institutional Review Board was not required because hospital discharge data are fully anonymous. For the same reason, informed consent is not possible and not required.**

**We have clarified the Methods in the revised manuscript to better describe the context of this research study in France and added the reference of the French law (Methods/Study design page 5 lines 165-168 of the “marked-up” revision).**

Throughout (including Tables and Figures): Please report p values as P<0.001 rather than P<0.0001.

**This has been changed throughout in the revised manuscript.**

Lines 191-193: Your study is observational, therefore please remove language that may imply causality (e.g., ‘effects of’ and ‘detrimental’). Refer instead to associations.

**We agree with the editor. We have removed language that may imply causality in Results and titles of Figures 2 and 3 (“associations” rather than “effects”).**

Line 198: Related to the above, please reword ‘exacerbated’, to avoid implying a causal relationship.

**Please see above.**

Please define the dashed lines in the key for Figure 1.

**We have added a legend in Figure 1.**

The data labels in Figures 2 and 3 are difficult to read, please enlarge the text slightly.

**Following PloS Medicine’s recommendations, we have changed all fonts from Calibri to Arial in all 3 Figures of the revised manuscript. In addition, we changed “Predicted risk” and “Observed risk” into “Prediction” and “Observation” to gain space in the x-axis legend and improve readability. It seems all 3 Figures now pass the PACE tests and they are provided individually as TIF files with 300dpi resolution.**

Please define the error bars in Figures 2 and 3 in the figure legend or title.

**Error bars refer to 95% CI of risks. This has been added in the y-axis legend of Figures 2 and 3.**

Please remove the COI information from References 1, 5, 8, 16, 17, and 20 (please check for others).

**We have updated the Endnote template for PloS and this has been systematically corrected in the revised manuscript.**

Did your study have a prospective protocol or analysis plan? Please state this (either way) early in the Methods section.

a) If a prospective analysis plan (from your funding proposal, IRB or other ethics committee submission, study protocol, or other planning document written before analyzing the data) was used in designing the study, please include the relevant prospectively written document with your revised manuscript as a Supporting Information file to be published alongside your study, and cite it in the Methods section. A legend for this file should be included at the end of your manuscript.

b) If no such document exists, please make sure that the Methods section transparently describes when analyses were planned, and when/why any data-driven changes to analyses took place.

c) In either case, changes in the analysis-- including those made in response to peer review comments-- should be identified as such in the Methods section of the paper, with rationale.

**According to French laws, the study protocol was submitted to the Health Data Hub (registration number F20220215185433 delivered on 02/15/2022, available in French language at** [**https://www.health-data-hub.fr/projets/fard-oh**](https://www.health-data-hub.fr/projets/fard-oh)**). The approval of an Institutional Review Board was not required because hospital discharge data are fully anonymous. We have expanded the Methods/Study design to better describe the context of this research study in France (please, see above responses).**

**The main objective stated in our study protocol is to assess the unmeasured burden of alcohol use disorders and other mental disorders in France with use of the French National Hospital Discharge database. Regarding the present study focusing on COVID-19 inpatients, we had not pre-specified hypotheses. However, our prospective analysis plan was clearly: 1) to check if the results from the meta-analyses on the association between mental disorders and COVID-19-related mortality would hold true in a nationwide sample of all French COVID-19 inpatients; and 2) to assess the potential role of salvage therapy triage to explain this association. Following previous editors’ comments, the main study objectives have been stated more clearly in the Abstract/Background and Introduction (please, see above responses).**

**We did not change our analysis plan based on the study results. Obviously, the suggestion of the academic editor to conduct a causal mediation analysis and the numerous comments of reviewer #3 led to new analyses which are now described in the revised manuscript (causal mediation analyses: “Following the editor’s comment, we conducted…” in Methods/Statistical analyses page 6 lines 213-217 of the “marked-up” revision; Results page 8 lines 275-279 of the “marked-up” revision) or in the following point-by-point detailed responses to reviewer #3.**

**References**

Harris, E. C. and B. Barraclough (1998). "Excess mortality of mental disorder." Br J Psychiatry **173**: 11-53.

Vai, B., et al. (2021). "Mental disorders and risk of COVID-19-related mortality, hospitalisation, and intensive care unit admission: a systematic review and meta-analysis." Lancet Psychiatry **8**(9): 797-812.

Fond, G., et al. (2021). "Association Between Mental Health Disorders and Mortality Among Patients With COVID-19 in 7 Countries: A Systematic Review and Meta-analysis." JAMA Psychiatry **78**(11): 1208-1217.

## Reviewer #1

Thanks for the opportunity to review your manuscript. My role is as a statistical reviewer, so my review concentrates on the study design, data, and analysis that are presented. I have put general questions first, followed by queries relevant to a specific section of the manuscript (with a page/line reference).

The study uses routinely collected hospital data from France in a population of hospitalised patients with COVID-19, and assesses risk factors for mortality (with 120 days follow-up) and use of salvage therapy (respiratory support and ICU admission). The key focus is on differences in patients with and without mental disorders. This is estimate with multivariable logistic regression models, and the results extended to a gap analysis (where the outcome is predicted without the mental disorder variable) and the difference between predicted and actual is compared.

There is some discussion about the limitation of measuring exposures from hospital records, one thing I think that needs to be mentioned is that some of the covariates/risk factors are ones that vary temporally and by classifying patients with any record of these (e.g. depression) as having that exposure, it assumes that the patient is still affected at the time of the COVID-19 admission. This might be best approached by shifting some of the language, e.g. 'patients with recorded history of depression'.

**We agree with the reviewer, although the remitting/relapsing profile of chronic conditions is not specific to some categories of mental disorders and may apply to several risk factors of severe COVID-19 (e.g., smoking) and somatic comorbidities (e.g., stroke). In this sense, another reviewer asked for a sensitivity analysis on the effects of the time window used to define covariates. We found reassuringly that the main study results were robust to a left-truncation of all discharge diagnosis data recorded before Jan 1, 2019, although the magnitude of the problem (i.e., the prevalence rate of pre-existing mental disorders) would be unduly underestimated (please, see below our detailed response to comment #4 of reviewer #3).**

**Following the reviewer’s comment, we added “pre-existing” in many instances (in particular in the Tables and titles of Figures) to remind the definition of all covariates in the revised manuscript.**

This is getting out of my area of expertise, but I was not certain about the classification of Down's syndrome as purely a 'mental disorder'. This is usually classified primarily as a genetic disorder that commonly presents with both mental and physical disabilities, rather than a purely mental disorder.

**We agree with the reviewer that including Down’s syndrome among mental disorders may be controversial, although they have in common stigmatization and higher risk of premature death (Harris et al. Br J Psychiatry 1998). We selected mental disorders based on previous meta-analyses on the associations of mental disorders with COVID-19-related mortality (Vai et al. Lancet Psychiatry 2021, Fond et al. JAMA Psychiatry 2021). Both meta-analyses included Down’s syndrome either explicitly as a search term (Vai et al. Lancet Psychiatry 2021) or implicitly by selecting “studies that included participants with a diagnosis of mental health disorders according to the DSM or any version of the International Classification of Diseases (ICD)" (Fond et al. JAMA Psychiatry 2021). Regarding ICD-10 used to code discharge diagnoses in France, its Chapter V “Mental and behavioral disorders“ (F00-F99) includes “Mental retardation” (F70-F79). While Down's syndrome (Q90) is classified in Chapter XVII “Congenital malformations, deformations and chromosomal abnormalities” (Q00-Q99), these patients are also often recorded with F7x codes to characterize the level of mental retardation. In our study, 222 (20.9%) of 1122 patients recorded with Q90 were also recorded with F7x codes.**

**We clarified in the revised manuscript that “we identified pre-existing mental disorders based on previous meta-analyses” (Methods/procedures page 6 line 190 of the “marked-up” revision).**

P3, L94. I went to look at the information about the method of record linkage, and the website was in French (alas, French was not a study option for me in high school). With an extra sentence or two of detail would you be able to specify the record linkage method? E.g. was it a statistical linkage key? What were the identifiers used? Was it encrypted?

**The method of hospital record linkage is not probabilistic but directly based on individual identifiers that are similarly encrypted in all French public or private hospitals. Individual identifiers are based on unique French Social Security numbers (13 digits) that involve gender (1 digit), month and year of birth (4 digits), place of birth (5 digits), and a sequential number (3 digits), with an additional validation key of the Social Security number (2 digits). The French Social Security is the unique, primary payer of healthcare in France, and no hospital bill can be reimbursed if any of multiple controls fail. The first control is about the French Social Security number that is first validated with the validation key by hospital administration and then similarly encrypted in all hospitals when generating the hospital bill (this is the “unique anonymous identifier” available in the national hospital discharge database). Given the strong financial incentives for hospitals, the rate of linkage between hospital stays (bills) is extremely high (above 99.9%). Furthermore, we conservatively selected patients residing in metropolitan France (i.e., foreigners may have a weaker Social Security number due to a smaller number of countries of birth (<200 country codes) compared to municipalities of birth (>30 000 municipality codes), and they are also more likely to be lost in the follow-up) and we did not consider scarce hospital bills that failed controls other than Social Security number (they are reported in the database).**

**Following the reviewer’s comment, we completed “Unique anonymous identifiers” with “based on encrypted Social Security numbers” (Methods page 5 line 151 of the “marked-up” revision).**

P4, L102. Just wondering about the terminology, as the data is specifically from human participants. Is the law about research where human participants are not directly contacted?

**Yes, this French law has been specifically ordered for research studies involving secondary analyses of the national hospital discharge database. Accordingly, the approval of an Institutional Review Board was not required because hospital discharge data are fully anonymous. For the same reason, informed consent is not possible and not required. It is actually forbidden by this law to try to re-identify (and possibly contact) any anonymous patient recorded in the database.**

**We have clarified the Methods/Study design to better describe the context of this research study in France and added the reference of the French law (Methods page 5 lines 160-168 of the “marked-up” revision).**

P4, L110. Was mortality recorded in the hospital records or taken from record linkage with national mortality records? Is there any way to check if most mortality was capture from hospital records, or is it possible that a significant amount of mortality outside of hospitals after separation was not captured?

**Only in-hospital mortality is available in the national hospital discharge database. We purposely conducted the main analysis with use of a 120-day follow-up to capture more broadly COVID-19-related deaths after the first hospital discharge.**

**We formerly reported the place of COVID-19-related deaths in 2020-2021 from French medical cause-of-death certificates (please see Discussion/limitation section). Most of these deaths occurred at hospital (70.4%, with concordant total numbers between our national study and this other national source) and otherwise in nursing homes (23.3%). Given the high proportion of people living with dementia in nursing homes, it further supports that patients with mental disorders had lower access to life-saving measures (admission at acute hospitals in the case of nursing homes) and higher mortality risks.**

P4, L124. To clarify, if someone had one of the disorders that define the main exposure in the previous 8 years they were assumed to still have the disorder at the time of the COVID-19 admission? This seems reasonable for some of the disorders (e.g. dementia) but some of these disorders (e.g. depression, alcohol) might not be present at the time of admission of interest, i.e. it is assumed that there is no variation in the disorder over time.

**Please see our response above and addition of “pre-existing” throughout the revised manuscript.**

P5. L141. Very pleased to see that you have selected a key set of variables and not done a variable selection process - with the entire French population you have plenty of sample size to use all the covariates of interest. What criteria was used to select the particular covariates from the multitude of potential ones available in the hospital data?

**Thank you! As for mental disorders, we based our selection of covariates on previous meta-analyses (early-identified risk factors of severe COVID-19) and results of large hospital databases (severe comorbidities of the Charlson Comorbidity Index and transplant status) (please, see references cited in Methods/procedures). We agree with the reviewer that the associations of additional chronic diseases could be of interest and added to the multivariate model. However, this was not the focus of our study. Of note, we have worked over years on this very large database and found generally that the addition of a new covariate has usually minimum impact on the strengths of associations found previously (when the model was already well adjusted with many covariates).**

P5, L144. This is an interesting approach that reminds me of a standardised mortality ratio. Is it possible for the full details of this procedure to be include either with reference to a publication with the details in it or with the addition of the material into an appendix? This is important for the peer review process.

**Thank you again! The reviewer is correct as we relied on usual practices to compare the performance of hospitals with use of Hospital Standardized Mortality Ratios (HSMR) based on individual data (Roalfe et al. BMC Health Serv Res 2008, Jacques et al. BMC Health Serv Res 2013, Garcia-Torrecillas et al. BMC Cancer 2019). The basics of this method are (there are many variants): 1) to assess the associations of several main descriptors of hospital case-mix (e.g., gender, age, type of admission, comorbidities) and mortality in the whole sample with use of a multivariate logistic model (that may be also stratified on Diagnosis-Related Groups DRG); 2) to calculate expected deaths in each hospital as the (DRG-weighted) sum of individual probabilities of death; and 3) to compute HSMR as expected/observed deaths for each hospital.**

**Our statistical approach is similar, although adapted to our study setting and objectives. In this regard, we assumed that COVID-19-related mortality risk is primarily explained by the vulnerability level of COVID-19 inpatients (e.g., older age and severe comorbidities) and the intensity of COVID-19 caseload surges in hospitals (i.e., with higher triage for life-saving resources at hospital). The first set of analyses comforted both assumptions (Table 1).**

**In the second set of analyses discussed here, we aimed at further disentangling the impact of these two main effects in the subgroup of COVID-19 patients with mental disorders who had expectedly higher vulnerability level (Introduction; Tables S4 and S5) and may have had lower access to salvage therapy during COVID-19 caseload surges in hospitals (Introduction; Table 2). Accordingly, we considered patients without mental disorders admitted during the first inter-wave period as the reference population with the best prognostic outcomes (Table 1). The logistic model was estimated in this reference population (c statistic of 0.846), with expectedly the same means calculated for observed and predicted mortality risks (Figure 2). The same logistic model was applied to all other patients to assess individual probabilities of death expected at baseline (compared to the reference population) and standardized means by subgroup. This approach originally allowed exhibiting (Figure 1): 1) the decrease of the baseline mortality risk over time irrespective of mental disorder status (in accordance with raw data on vulnerability level assessed by pandemic periods; Results/2^nd^ paragraph, Table S1); 2) the impact of COVID-19 caseload surges in hospitals leading to excess mortality risks (i.e., difference between observed and predicted mortality risks) irrespective of mental disorder status; and 3) the impact of mental disorders with higher excess mortality risks in all pandemic periods. We used the same approach for the two main outcomes to contrast the impact of these two main effects on mortality and access to salvage therapy and conducted multiple sensitivity analyses to assess the robustness of the study results.**

**We have clarified this second set of analyses and added previous references on HSMR in the revised manuscript (Methods page 6 lines 218-235 of the “marked-up” revision).**

I also couldn't see in the manuscript where p-values from the t-test procedure where applied. Is this done across the entire dataset for actual vs. predicted outcomes, with a binary outcome? If binary then a McNemar's test would probably be appropriate than a test intended for a continuous variable. Given the sample size though I think the CI is much more useful for inference.

**The observed outcome is binary and the individual predicted outcome is a probability. In agreement with the reviewer about the large sample size and usual practices to compute HSMR based on sums of individual probabilities, we computed sample means for all patient groups and tested mean differences with use of paired t-tests.**

Table S1/S2 (and similar tables throughout the appendix). Given the large sample size and descriptive nature of the tables I think the p-value is superfluous.

**We agree with the reviewer on a statistical ground, though we would prefer to keep up with medical traditions.**

**References**

Harris, E. C. and B. Barraclough (1998). "Excess mortality of mental disorder." Br J Psychiatry **173**: 11-53.

Vai, B., et al. (2021). "Mental disorders and risk of COVID-19-related mortality, hospitalisation, and intensive care unit admission: a systematic review and meta-analysis." Lancet Psychiatry **8**(9): 797-812.

Fond, G., et al. (2021). "Association Between Mental Health Disorders and Mortality Among Patients With COVID-19 in 7 Countries: A Systematic Review and Meta-analysis." JAMA Psychiatry **78**(11): 1208-1217.

Roalfe, A. K., et al. (2008). "Standardisation of rates using logistic regression: a comparison with the direct method." BMC Health Serv Res **8**: 275.

Jacques, R. M., et al. (2013). "Did hospital mortality in England change from 2005 to 2010? A retrospective cohort analysis." BMC Health Serv Res **13**: 216.

Garcia-Torrecillas, J. M., et al. (2019). "Spatial and temporal variations in Spain in the standardised ratio of in-hospital mortality due to colorectal cancer, 2008-2014." BMC Cancer **19**(1): 310.

## Reviewer #2

The manuscript submitted by Dr. Schwarzinger et al. aims to analyze the association between mental disorders and COVID-19 related life-saving measures and mortality. The paper is very clearly written and of great quality. Authors use an impressive and exhaustive hospital dataset which enables them to go further previous studies. Their main finding is that COVID-19 patients with mental disorders had lower odds in access to ICU during the pandemic in France and had higher mortality rates.

**Thank you very much!**

Major comments:

- Statistic procedures: the authors state that 'we considered that patients without any mental disorder during the first inter-wave period had the best prognostic outcome'. What if this assumption is broken? It would be good to have a sensitivity analysis on this, and discuss the choice of the reference period, especially given that all results are based on this analysis.

**Thank you for your comment as we realized that the description of this second analysis was not detailed enough. Indeed, this second set of analyses was based on the first set of analyses showing that patients without any mental disorders during the first inter-wave period had the best prognostic outcome in this study sample (Table 1).**

**We have corrected this in the revised manuscript by adding “based on previous findings” (page 6 line 222 of the “marked-up” revision).**

- Similarly, the rate of testing in France changed dramatically over the course of the pandemic, and tests were still not fully available in the first inter-wave period (see raw data here: https://www.ecdc.europa.eu/en/publications-data/covid-19-testing), with large delays between test and results in July-August 2020. Would test availability have an impact on the results?

**We don’t believe so. Our study sample is based on all inpatients recorded with symptomatic COVID-19 in mainland France. PCR COVID-19 tests were only available at hospital at the beginning of the pandemic (Haut Conseil de la Santé Publique March 10, 2020) and testing capacity increased rapidly over time in all hospitals. More importantly, it is very unlikely that patients with symptomatic COVID-19 in ambulatory care and deemed severe enough for hospital admission were left undiagnosed at hospital.**

Minor comments:

- Line 67: it would be good to specify which 'specific categories' were inconclusive in other papers.

**We agree with the reviewer and we have clarified which categories were significantly associated with prognosis (mood disorders, schizophrenia) in the revised manuscript (Introduction page 4 line 114-117 of the “marked-up” revision).**

**References**

Haut Conseil de la Santé Publique (March 10, 2020). Provisional statement: Patients at risk of severe forms of Covid-19 and prioritising access to diagnostic tests [available from: <https://www.hcsp.fr/Explore.cgi/AvisRapportsDomaine?clefr=798>].

## Reviewer #3

This is a review of the manuscript "Mental disorders and COVID-19-related life-saving measures and mortality: a nationwide, retrospective hospital cohort study over an 18-month period in France" submitted for publication in PLOS Medecine. This manuscript addresses a timely, important and debated research question, i.e. whether mental disorders are independently associated with COVID-19-related mortality and access to salvage therapy. However, the current submission may suffer from several methodological and statistical issues that weaken its conclusion, but most issues could be relatively easily addressed. Below are several suggestions that I hope the authors will find useful.

**Thank you for your overall positive assessment! Please find below our responses to address your comments and changes made to improve the manuscript.**

1/ Introduction: "Mental disorders also seem to increase the risk of serious COVID-19 outcomes, especially mortality, as two systematic reviews and meta-analyses have suggested". To the reviewer's knowledge, this point is debated. Two recent studies taking into account many confounders, and in particular medical comorbidities, found that respectively psychiatric disorders (doi: 10.1038/s41380-021-01393-7) and major depression (doi: 10.1371/journal.pone.0255427) could be significantly associated with reduced mortality, possibly due to the potentially protective role of certain psychotropic medications (PMID: 34608263). Because of this debate, it would be important to detail and shortly discuss both the measures of associations and the prevalence rates of mental disorders in those meta-analyses. Indeed, the prevalence rates of major psychiatric disorders in Barcella et al. (PMID: 33894064) and Vai et al. (PMID: 34274033) meta-analytic studies were very low (3.6% and 3.1%, respectively), which may suggest that a small proportion of people with psychiatric disorders, those with important medical comorbidities, have increased risk of COVID-19-related mortality.

**We agree with the reviewer that previous meta-analyses may not provide definite conclusions (“seem” and “suggested” in the quoted sentence) as summary statistics of unadjusted associations were highly heterogeneous (Vai et al. Lancet Psychiatry 2021, Fond et al. JAMA Psychiatry 2021). In agreement with the reviewer, we previously developed that “inconsistent and often insufficient adjustment” was a major problem in the underlying studies (first paragraph of the Introduction).**

**However, several main factors other than adjustment may contribute to heterogeneous results such as the study setting, scope and definition of mental disorders (Vai et al. Lancet Psychiatry 2021, Fond et al. JAMA Psychiatry 2021). Most studies were conducted in the community rather than at hospital (16/21 studies in (Vai et al. Lancet Psychiatry 2021); 14/16 studies in (Fond et al. JAMA Psychiatry 2021)), while pre-existing mental disorders were significantly associated with an increased risk of hospital admission (pooled adjusted OR: 1.76 [95% CI, 1.05-2.96] (Vai et al. Lancet Psychiatry 2021)). Accordingly, the prevalence rate of mental disorders should expectedly be higher in our study as well as in the two hospital-based studies cited by the reviewer (Hoertel et al. Mol Psychiatry 2022, Gunster et al. PLoS One 2021) compared to previous meta-analyses. Of note, the study of Barcella et al. is not a meta-analysis but a population-based study conducted on all Danish people diagnosed with COVID-19 in 2020 (Barcella et al. Acta Psychiatr Scand 2021).**

**Regarding the scope and definition of mental disorders, it is unclear whether these two cited studies would alter previous meta-analytical results. In the first study (Hoertel et al. Mol Psychiatry 2022), 49 089 COVID-19 patients were identified in academic hospitals of Ile-de-France region during the first wave of COVID-19 pandemic. Mental disorders encompassed the whole ICD-10 chapter of “mental and behavioral disorders” (F01-F99) and were assessed from discharge diagnoses of the first COVID-19 hospitalization. Such a broad scope may blur (positive or negative) independent associations of different categories of mental disorders with mortality. Moreover, mental disorders may have been underestimated by using only discharge diagnoses of the first COVID-19 hospitalization, with potential misclassification bias in the study results (see our detailed response below to comment #4).**

**In the second study (Gunster et al. PLoS One 2021), 8769 COVID-19 patients were identified from the hospitals of a German sickness fund. Depression (along other Elixhauser comorbidities) and cognitive disorders were the only two categories of mental disorders assessed with use of discharge diagnoses of the first COVID-19 hospitalization. The final multivariate logistic model was based on variable selection (cognitive disorders were not retained) and the lower association of depression with 180-day mortality may be insufficiently controlled for. Most importantly, as in the first study, mental disorders may have been underestimated by using only discharge diagnoses of the first COVID-19 hospitalization.**

**We expanded the presentation of past research in the revised manuscript (including the first reference cited by the reviewer) (Introduction: page 4 lines 106-117 of the “marked-up” revision).**

Methods and statistics:

2/ Population selection: Why excluding all patients discharged alive after day-case admission or recorded with asymptomatic SARS-CoV-2 infection. If there is a significant difference in the prevalence of psychiatric disorders in this subsample compared to the one used in the analysis, it may introduce a selection bias. In addition, for patients hospitalized (even for several hours) for several health conditions, it may be difficult to distinguish the specific contribution of SARS-CoV-2 infection, for which the symptoms and its impact were still not completely known. Finally, patients with COVID-19 and a day-case admission could have received oxygen therapy. Therefore, I strongly suggest including in the main analysis all these patients with a COVID-19 diagnosis record to avoid such potential selection bias. Sensitivity analyses excluding these patients can be presented as supplementary analysis.

**We disagree with the reviewer’s suggestion. Previous meta-analysis showed that mental disorders were significantly associated with increased risks of hospital admission (pooled adjusted OR: 1.76 [95% CI, 1.05-2.96]) and mortality (pooled adjusted OR: 1.31 (95% CI, 1.13-1.51)) (Vai et al. Lancet Psychiatry 2021). The same positive direction of associations suggests higher severity of COVID-19 in patients with pre-existing mental disorders. In this regard, it is unclear whether the subsample of patients discharged alive after day-case admission or recorded with asymptomatic SARS-CoV-2 infection (92 573 (16.6%) of 558 323 patients in our study) reflects COVID-19 severity or heterogeneous hospital care practices.**

**PCR COVID-19 tests were only available at hospital at the beginning of the pandemic. Despite official guidelines to prioritize PCR COVID-19 tests for symptomatic patients at risk of severe COVID-19 (Haut Conseil de la Santé Publique March 10, 2020), we found that the proportion of this subsample varied largely across the 1187 French acute hospitals from 0 to 100% with outstanding hospital care practices at the academic hospitals of Marseille (23 125 (74.4%) of 31 071 patients). It suggests that admission criteria of this subsample were multifactorial including PCR testing capacity at hospitals (academic vs. non-academic) and considerations other than COVID-19 severity (for instance, enrolment in research studies at the academic hospitals of Marseille).**

**As our main objective was to study the association of pre-existing mental disorders with mortality, we conservatively excluded this subsample to avoid “noisy” data and focus on the more homogeneous population that is indeed at high mortality risk (including the most severe COVID-19 symptomatic patients who died within the first day of hospital admission).**

3/ Mortality attributable to COVID-19 versus to other causes? Since the authors are interested in COVID-19-related deaths, the 120-day mortality may not be appropriate. According to the London Imperial College (https://www.imperial.ac.uk/), the mean delay between infection onset and death is 16.0 (SD= 8.2) days. Using 120-day mortality, it is highly probable that a substantial proportion of deaths is not attributable to COVID-19. Given that people with versus without mental disorders are more likely to die (form cardiovascular disorders, suicide, etc…) (doi:10.1192/bjp.173.1.11), the results may wrongly attribute this excess mortality to COVID-19. My recommendation would be to use 28-day mortality in the main analysis, as usually done in RCTs, to address this point, and modify accordingly Table 1.

**As formerly stated in the Discussion/limitation section, we agree with the reviewer that “the main limitation of our study points to the assessment of COVID-19-related mortality”. COVID-19-related mortality is hard to define, especially with respect to time frames when a death should be attributed to COVID-19. In COVID-19 RCTs, most of the drug effect was expected shortly as increased follow-up may unduly increase delays to market effective drugs (and trial costs), and the usual cut-off at 28 days in RCTs cannot be taken as a general rule for observational studies. On the contrary, the long term effects of COVID-19 are of current research interest, in particular in patients admitted to hospital and ICU (see the review conducted in the hospital-based study previously cited by the reviewer (Gunster et al. PLoS One 2021)).**

**In addition, patients with mental disorders have higher risks of “premature” death from natural and unnatural causes as shown in the seminal work of Harris and Barraclough cited by the reviewer (Harris et al. Br J Psychiatry 1998). We purposely addressed this issue by disentangling the effects of patient vulnerability and COVID-19 caseload surges in hospitals on the outcomes of COVID-19 patients with pre-existing mental disorders and found that both mortality risks predicted at baseline (in relation to higher vulnerability level) and excess mortality risks (due to COVID-19 caseload surges) were significantly higher in patients with pre-existing mental disorders compared to others (Figure 2). .**

**As the reviewer may not have noticed, we previously reported (Methods, Results, and Discussion/limitations) that the main study results using 120-day mortality (Figure 2) were robust in a sensitivity analysis using 28-day mortality (Figure S10). The in-depth comparison of the two sets of results is informative on the comment of the reviewer:**

**1) Baseline mortality risks: patients with pre-existing mental disorders had systematically higher baseline mortality risks compared to others at 28 days (14.0% [95% CI, 13.9-14.1] and 8.9% [95% CI, 8.8-8.9], respectively) and 120 days (21.2% [95% CI, 21.0-21.4] and 13.3% [95% CI, 13.2-13.4], respectively). The differences in baseline mortality risks between the two groups assess the effect described by Harris and Barraclough. This differences slightly increased over time (+5.1% at 28 days, +7.9% at 120 days) and it may suggest that misclassification bias increases over time in patients with mental disorders. However, it should be noted that any of those “COVID-related deaths” may not be attributable to COVID-19 but rather to the underlying vulnerability of COVID-19 patients, irrespective of patients’ group and timing of mortality assessment.**

**2) Excess mortality risks: patients with pre-existing mental disorders had also systematically higher excess mortality risks compared to others at 28 days (+9.7% [95% CI, 9.3-10.2] and +5.0% [95% CI, 4.7-5.2], respectively) and 120 days (+9.3% [95% CI, 8.9-9.8] and +5.1% [95% CI, 4.9-5.4], respectively). Excess mortality risks were the same at 28 and 120 days in each group pointing to an early cause for in COVID-19 hospital management that is unrelated to patients’ vulnerability. In this regard, other analyses on salvage therapy (Table 2, Figure 3) strongly suggest that patients with pre-existing mental disorders were more frequently denied beneficial salvage therapy despite higher needs.**

**We have now expanded the Discussion/limitation section in the revised manuscript to provide more feedback on the implications of this sensitivity analysis at 28 days (Discussion page 11 lines 388-392 of the “marked-up” revision).**

4/ Mental disorders assessment: The 9-year timeframe for assessing mental disorders is very discussable, as for example people who had 1 major depressive episode 7 years ago but no depressive symptoms for the last 5 years cannot be considered as having a diagnosis of psychiatric disorder. Given the extremely high prevalence of psychiatric disorders reported in this study (33%), it suggests that most people "with mental disorders" have a past history but not a current diagnosis of psychiatric disorders, limiting the interpretability of the findings. A more conservative 1-year history of psychiatric disorders before AND NOT during the hospitalization with/for COVID-19 would clarify the link between preexisting "active" psychiatric disorders and COVID-19-related mortality. Importantly, mental disorders should not be assessed based on the diagnosis record of the hospitalization with/for COVID-19, since COVID-19 is significantly associated with increased incidence of neurological or psychiatric diagnoses (PMID: 33836148), and these patients with mental disorders related/consecutive to COVID-9 are more likely to have had severe COVID-19, and consequently increased risk of death. To avoid this bias, I suggest to distinguish 3 groups of patients: i) with a past-year diagnosis of mental disorder (excluding the hospitalization with COVID-19), (ii) no 9-year history of mental disorders, and (iii) incident diagnosis of psychiatric disorders (during the hospitalization and not in the past 9 years). This analysis would help distinguish the true effect of pre-existing current psychiatric disorders from that of secondary psychiatric disorders, such as frequently seen in ICUs, which may simply reflect severe COVID-19.

**We agree with the reviewer that the definition of mental disorders including the timing of assessment may be critical in the study results. However, we are somewhat puzzled with the line of reasoning and suggestions of the reviewer who may not be familiar with national discharge diagnosis databases.**

**Firstly, ICD-10 discharge diagnoses are merely indicating the presence of a chronic condition during a given hospitalization. Most frequently, the label and definition of ICD-10 codes used to define chronic conditions do not involve temporality and it cannot be inferred that the chronic condition was incident or prevalent at hospital entry. Accordingly, this distinction can only be made with some certainty when a (prevalent) chronic condition was recorded in hospitalizations preceding the index hospitalization (e.g., the first hospitalization with a COVID-19 diagnosis in our study).**

**Secondly, besides the primary discharge diagnosis that should be coded (e.g., usually COVID-19 for the index COVID-19 hospitalization in our study), associated discharge diagnoses are knowingly under-coded compared to medical charts’ reviews (Quan et al. Health Serv Res 2008). In the exceptional context of COVID-19 overload surges at hospital, under-coding of associated discharge diagnoses is even more likely for the index COVID-19 hospitalization. Accordingly, reliance on previous hospitalizations seems even more important to define a prevalent chronic condition. For instance, in the national Danish study of Barcella et al. cited by the reviewer (see above comment #1), mental disorders were tracked back until 1994 (Barcella et al. Acta Psychiatr Scand 2021). In a study of the UK biobank, mental disorders were tracked back until 1981 (Yang et al. Lancet Healthy Longev 2020).**

**Thirdly, the reviewer suggests that “mental disorders should not be assessed based on the diagnosis record of the hospitalization with/for COVID-19, since COVID-19 is significantly associated with increased incidence of neurological or psychiatric diagnoses”. This suggestion seems at odd with the two hospital-based studies supporting the argumentation of the reviewer as both studies only relied on hospital records during the index COVID-19 hospitalization (Hoertel et al. Mol Psychiatry 2022, Gunster et al. PLoS One 2021). More importantly, the index COVID-19 hospitalization had a median (IQR) length of stay of 11 (6-19) days in our study sample and it seems unlikely that a chronic condition would develop because of COVID-19 in such a short timeframe. In the study of Taquet et al. cited by the reviewer (Taquet et al. Lancet Psychiatry 2021), mental disorders were assessed from EHR data over 6 months after the index COVID-19 diagnosis and the authors disregarded mental disorders recorded within the first 14 days in sensitivity analyses. In our study, we used a conservative approach to assess mental disorders to avoid reverse causation: “prognostic factors were assessed at the first date of recorded COVID-19 diagnosis at hospital with use of all discharge information recorded over the last nine years” (see Methods/procedures) and therefore we did not track new, possibly secondary (or previously under-coded) mental disorders recorded in hospitalizations following the index COVID-19 hospitalization.**

**Finally, we agree with the reviewer that mental disorders have different natural history profiles with some conditions that are usually irreversible (dementia; schizophrenia; Down syndrome; other learning disabilities) and other conditions that are usually marked by remitting/relapsing sequences (depression; anxiety disorders; alcohol use disorders; opioid use disorders). Regarding irreversible conditions, any record before/at index COVID-19 hospitalization logically defines a prevalent chronic condition. Regarding remitting/relapsing conditions, we agree with the reviewer that applying the same logic becomes debatable as time elapses before index COVID-19 hospitalization. However, it should be noted that all diagnoses were recorded at hospital (including psychiatric wards) and it may be assumed that we identified patients with more severe relapsing profiles compared to e.g., patients only cared in the ambulatory setting. In addition, such remitting/relapsing profile is not specific to mental disorders (smokers may quit and relapse; patients may fully recover from a stroke and have another one…) and the same time window should be used to define all chronic conditions.**

**To address the reviewer’s concern and taking into account previous considerations, we looked further at the possible effects of a shorter time window to define all chronic conditions in our study. All discharge diagnoses were left-truncated at January 1, 2019. The results of this complementary analysis are threefold:**

**1) The prevalence of pre-existing mental disorders decreased from 153 870 (33.0%) in the main analysis to 118 080 (25.4%) in the 465 750 patients. All categories of mental disorders remained mostly (>50%) recorded before the index COVID-19 hospitalization (>70% in the main analysis). The reviewer may still be concerned about “the extremely high prevalence of psychiatric disorders in this study”. However, this sensitivity analysis merely points the underestimation of the prevalence rate of pre-existing mental disorders when hospital-based studies are only based on discharge diagnoses recorded during the index COVID-19 hospitalization (Hoertel et al. Mol Psychiatry 2022, Gunster et al. PLoS One 2021).**

**2) Similar independent associations of pre-existing mental disorders with 120-day mortality were found in the main analysis (Table 1) and this sensitivity analysis (see Table below), except for dementia. The prevalence of dementia decreased from 67 539 (14.5%) in the main analysis to 60 182 (12.9%) in the sensitivity analysis. Because of left-truncation of information at Jan. 1, 2019, about 7400 patients with dementia recorded before 2019 were under-diagnosed at index COVID-19 hospitalization. These patients had expectedly a more severe form of dementia (and higher mortality risk) compared to patients recorded after 2019 and the reversal of association suggests that a too narrow time window to define chronic (at least irreversible) conditions may lead to misclassification bias.**

| **Dementia** | **0.96 (0.94-0.98)** | **0.0002** |
| --- | --- | --- |
| **Depression** | **1.03 (0.99-1.06)** | **0.1838** |
| **Anxiety disorders** | **1.07 (1.03-1.11)** | **0.0009** |
| **Schizophrenia** | **1.10 (1.04-1.17)** | **0.0005** |
| **Alcohol use disorders** | **1.11 (1.07-1.16)** | **<.0001** |
| **Opioid use disorders** | **0.90 (0.78-1.04)** | **0.1490** |
| **Down syndrome** | **4.18 (3.56-4.92)** | **<.0001** |
| **Other learning disability** | **1.58 (1.45-1.74)** | **<.0001** |
| **Other disorders with psychiatric ward admission** | **1.23 (1.11-1.38)** | **0.0002** |

**3) Compared to the main analysis (Figure 2), baseline mortality risks significantly increased in the sensitivity analysis (Figure below) for both patient groups (with mental disorders: from 21.2% [95% CI, 21.0-21.4] to 22.2% [95% CI, 22.0-22.4]; without mental disorders: from 13.3% [95% CI, 13.2-13.4] to 14.3 [95% CI, 14.2-14.41]). It may be explained by the selection of more severe, “active” patients with mental disorders in the first group, while about 35 000 patients with pre-existing mental disorders (and more comorbidities) were removed from the first to the second group of patients. Consequently, excess mortality risk due to COVID-19 caseload surges in hospitals decreased in patients with mental disorders (+9.3% [95% CI, 8.9-9.8] and +8.4 [95% CI, 7.9-9.0], respectively) and was the same in patients without mental disorders (+5.1% [95% CI, 4.9-5.4] and +5.2 [95% CI, 4.9-5.4], respectively).**

**Altogether, these additional results support that a larger rather than shorter time window should be used to define pre-existing mental disorders. This methodological approach is in agreement with usual practices to rely on all available discharge diagnoses databases to define pre-existing mental disorders (please see above (Barcella et al. Acta Psychiatr Scand 2021, Yang et al. Lancet Healthy Longev 2020)). In addition, this sensitivity analysis illustrates that a too short time window (or relying on the sole index COVID-19 hospitalization data) would unduly lower the magnitude of the problem related to pre-existing mental disorders on several aspects. Accordingly, we did not change the presentation of our study results.**

5/ Adjusting for comorbidity: although the Charlson Comorbidity Index is described in the methods, this variable is not reported in the result section or in the tables. Because medical comorbidity is a central confounder, I think it would be important to add, beyond all individual diagnoses, the total number of comorbidities (categorized in quintiles for example) to examine the specificity of associations between individual disorders, such as psychiatric disorders, and mortality (doi: 10.1038/s41380-021-01393-7).

**The Charlson Comorbidity Index was not included in the multivariate logistics because we used each of its components as covariates. This methodological approach is in agreement with usual practices for discharge diagnosis databases because sample size is very large (compared to clinical studies where the Charlson index was originally used), model fitting can only be better (multiple comorbidities vs. one index categorized in x classes), and the level of associations of different somatic severe comorbidities with COVID-19-related mortality is of interest per se (internal validity of the study results).**

6/ Adjusting for BMI: because patients with mental disorders are more likely to have altered BMI and because CDC data indicates a J-shaped (nonlinear) relationship between continuous BMI and disease severity (PMID: 33705371), BMI should be studied with a greater number of degrees of freedom, as recommended by the CDC: i.e., underweight (<18.5 kg/m2), healthy weight (18.5-24.9 kg/m2 [reference]), overweight (25-29.9 kg/m2), obesity (30-34.9 kg/m2), obesity II (35-39.9 kg/m2), obesity III (35-39.9 kg/m2), obesity IV (40-44.9 kg/m2), and obesity V (≥45 kg/m2).

**The CDC study (Kompaniyets et al. MMWR Morb Mortal Wkly Rep 2021) is only one study among many others on this topic. Previous meta-analyses found conflicting summary results on the association of BMI or obesity with COVID-19-related mortality (Zhang et al. Sci Rep 2021, Mahamat-Saleh et al. BMJ Open 2021) but they all agree on the high level of heterogeneity in underlying study results. In the CDC study, the main results are presented without adjustment on comorbidities! In the sensitivity analysis adjusting on (only seven) somatic comorbidities (and no mental disorders), the association remained only significant for obese people above 40 kg/m2 (Kompaniyets et al. MMWR Morb Mortal Wkly Rep 2021). Such odd presentation may be explained by the political will of CDC agency to point to the severity of obesity in the US but also their reliance on different sources of data, i.e., EHR data for BMI vs. discharge diagnosis data for comorbidities. Both sources may be incomplete (e.g., only 238 out of 800 hospitals reporting both height and weight in EHR data were selected in the CDC study), although the missing not at random processes are most likely different across sources and may bias the study results.**

**Because of previous meta-analyses, we formerly included obesity (BMI>=30 kg/m2) as a covariate as it may be associated with COVID-19-related mortality. Our study results support that this is not the case after full adjustment on multiple somatic and mental comorbidities (Zhang et al. Sci Rep 2021).**

**To address the reviewer’s concern, we looked further at the possible effects of BMI in our study. The ICD-10 version used in French hospitals (ICD-10-FR) does not allow coding BMI with multiple categories as height and weight are not recorded, i.e., only 2 categories [30-40[ and >=40 kg/m2 could be reliably coded from 2012 onwards. We found that 25 882 (22.0%) of 117 385 obese patients in the study sample were recorded at least once with “morbid obesity” (>=40 kg/m2) over the last nine years before/at index COVID hospitalization. The main study results on the independent associations of categories of mental disorders with 120-day mortality (Table 1) were unchanged when using this BMI categorization:**

| **Dementia** | **1.03 (1.01-1.05)** | **0.0105** |
| --- | --- | --- |
| **Depression** | **1.07 (1.04-1.10)** | **<.0001** |
| **Anxiety disorders** | **1.04 (1.01-1.07)** | **0.0186** |
| **Schizophrenia** | **1.15 (1.10-1.21)** | **<.0001** |
| **Alcohol use disorders** | **1.15 (1.12-1.19)** | **<.0001** |
| **Opioid use disorders** | **0.96 (0.86-1.07)** | **0.4482** |
| **Down syndrome** | **4.44 (3.79-5.20)** | **<.0001** |
| **Other learning disability** | **1.73 (1.60-1.87)** | **<.0001** |
| **Other disorders with psychiatric ward admission** | **1.26 (1.15-1.38)** | **<.0001** |

**In addition, we found that only “morbid obesity” may be independently associated with mortality (>=40 kg/m2: aOR=1.14 [95% CI, 1.10-1.18]; [30-40[ kg/m2: aOR=0.89 [95% CI, 0.87-0.91]), in agreement with CDC’s sensitivity analysis.**

**As the key focus of our study is on differences in patients with and without mental disorders, we did not add this sensitivity analysis in the revised manuscript.**

7/ Additional potentially important confounders of the association between mental disorders and mortality could be (i) the total number of medications, known to increase mortality (PMID: 32909235), (ii) the lower vaccination rates in patients with mental disorders (PMID: 35753318), and (iii) a greater disease severity when hospitalized due to a greater mean delay between infection onset and hospitalization. If available, these variables should be included in the models; if not, this may constitute important limitations that should be acknowledged.

**These variables are not available in French discharge diagnosis databases.**

**The study of Brockhattingen et al. cited by the reviewer (Brockhattingen et al. Eur Geriatr Med 2020) showed an independent association of the total number of medications and mortality in geriatric patients (median age of 83 years) before the COVID-19 pandemic. We do not believe that the total number of medications would be an important confounder given the younger age in our study sample (median age of 72 years, Table 1) and full adjustment on multiple chronic conditions (that are usually treated).**

**The study of Watson et al. cited by the reviewer (Watson et al. Lancet Infect Dis 2022) is a mathematical model showing the benefits of COVID-19 vaccination over time worldwide (without any focus on mental disorders). The study results of Watson et al. are in agreement with the decreasing trend found in the number of COVID-19 inpatients in 2021 for France (Figure 1). We agree with the reviewer that patients with mental disorders may have lower vaccination rates compared to others (Curtis et al. Br J Gen Pract 2022). However, we do not believe that vaccination status would be an important confounder as we found similarly in all pandemic periods that baseline and excess mortality rates were higher in patients with pre-existing mental disorders compared to others (Figure 2).**

**We agree with the reviewer that patients with mental disorders may have increased delay from infection onset to hospitalization compared to others and it may be associated with higher COVID-19 severity at hospital entry, although we did not find supporting evidence in the literature. We formerly conducted a sensitivity analysis on the subgroup of 371 016 (79.7%) inpatients recorded with COVID-19 respiratory symptoms who had a more severe form of COVID-19 on average (Table 1). Similar results were found (Figures S5-S6, Results), suggesting that increased delays would not be an important confounder.**

8/ "To limit residual confounding from multiple other severe somatic conditions, we assessed the delay between the latest acute hospital discharge for any reason other than pregnancy and psychiatry and first COVID-19 diagnosis record": it should be better explained how this reduces the residual confounding; in addition, not taking into account hospital discharge for psychiatric disorder may introduce a bias since the reason of hospitalization in Psychiatry is not so rarely for both psychiatric and non-psychiatric reasons (e.g., dementia with behavioral problems due to non-psychiatric reasons).

**Patients may be tracked in the national discharge diagnosis database for multiple conditions other than those commonly associated to COVID-19-related mortality and used in our study. Therefore, we added a tracker of any hospitalization before/at index COVID-19 hospitalization to additionally control for this unmeasured additional burden of disease. Previous admissions in psychiatry were not included in the tracker as they defined the by-default category of mental disorders unrecorded in other categories. While we agree that patients with mental disorders are often cared for non-psychiatric disorders in psychiatric wards, they are generally transferred to acute hospitals when non-psychiatric disorders are severe enough.**

9/ Tables 1 and 2 should also provide descriptive statistics for people alive.

**Descriptive statistics for people Alive can be easily computed as the difference between Total and Deceased columns.**

10/ Table 1. The AOR adjusted for "any mental disorder" should be provided as it corresponds to the main result of the study. The difference for any mental disorder (33% versus 30.5%) does not look very substantial and may not even be significant.

**In our opinion, “any mental disorder” is a too broad definition that may blur (positive or negative) independent associations of categories of mental disorders with mortality (see above response to comment #1). Accordingly, we assessed each of these categories in the multivariate model. As most (at least all frequent) categories were associated with higher mortality (Table 1), we further scrutinized the association of each category of mental disorders (Table S6) and “any mental disorder” (Figure 2) with higher mortality risk regarding predicted baseline and excess mortality risks (see above response to comment #4). These two analyses and their counterpart for salvage therapy (Table 2, Figure 3) provide much more insights into the mechanisms at stake compared to a basic multivariate logistic with “any mental disorder” as the only covariate.**

**It seems the reviewer is comparing the % of deaths at 120 days among patients with mental disorders (second column: 33.0%) with the % of mental disorders recorded among the Total sample (first column: 30.5%). The raw percentage of deaths at 120 days among patients with mental disorders is obviously higher when compared to that observed in the Total sample (second column, first line: 22.3%).**

11/ Details about the main multivariable logistic regression models for "any mental disorder" should be provided in supplementary material: list of variables included in the models (all covariates should be included), number of degrees of freedom, quality checks with residuals and collinearity diagnostics.

**This was not raised by the statistical reviewer #1. Collinearity is a priori not an issue in our study as covariates assess different concepts that are not overlapping. In addition, all algorithms converged confirming that collinearity was not issue in our study findings.**

**Collinearity may be an issue in mixing different sources of data, e.g., in the study of Hoertel et al. cited above by the reviewer where smoking status was more precisely assessed from EHR data, although it seems that discharge diagnoses related to smoking status were not removed from the broad definition of mental disorders (“F01-F99” includes F17 “Mental and behavioural disorders due to use of tobacco) (Hoertel et al. Mol Psychiatry 2022).**

12/ Given the apparently substantial and robust association between mental disorders and lower access to salvage therapy, it would be very interesting to perform a supplementary analysis testing the association between any mental disorder and mortality, while adjusting for all potential confounders listed in Table 1 as well as salvage therapy rate. A potentially non-significant or a reversed association would be interesting to discuss as it may help reconcile all prior findings highlighted in 1/

**The statistical approach suggested by the reviewer is inappropriate for correlated outcomes (Table S3). Following the editors’ suggestion, we have added a series of causal mediation analyses in the revised manuscript that do also support that pre-existing mental disorders have a direct effect on higher mortality risk that is not due to mediation or interaction with salvage therapy.**

13/ I would shortly discuss the potential impact of multiple testing on the results.

**Given the large sample size, the statistical reviewer #1 even suggested to remove p-values from the Tables. Most statistical tests led to p-values <10^-6^ and accounting for multiple testing is unlikely to change statistical significance.**

**References**

Vai, B., et al. (2021). "Mental disorders and risk of COVID-19-related mortality, hospitalisation, and intensive care unit admission: a systematic review and meta-analysis." Lancet Psychiatry **8**(9): 797-812.

Fond, G., et al. (2021). "Association Between Mental Health Disorders and Mortality Among Patients With COVID-19 in 7 Countries: A Systematic Review and Meta-analysis." JAMA Psychiatry **78**(11): 1208-1217.

Hoertel, N., et al. (2022). "Comorbid medical conditions are a key factor to understand the relationship between psychiatric disorders and COVID-19-related mortality: Results from 49,089 COVID-19 inpatients." Mol Psychiatry **27**(3): 1278-1280.

Gunster, C., et al. (2021). "6-month mortality and readmissions of hospitalized COVID-19 patients: A nationwide cohort study of 8,679 patients in Germany." PLoS One **16**(8): e0255427.

Barcella, C. A., et al. (2021). "Severe mental illness is associated with increased mortality and severe course of COVID-19." Acta Psychiatr Scand **144**(1): 82-91.

Haut Conseil de la Santé Publique (March 10, 2020). Provisional statement: Patients at risk of severe forms of Covid-19 and prioritising access to diagnostic tests [available from: <https://www.hcsp.fr/Explore.cgi/AvisRapportsDomaine?clefr=798>].

Harris, E. C. and B. Barraclough (1998). "Excess mortality of mental disorder." Br J Psychiatry **173**: 11-53.

Quan, H., et al. (2008). "Assessing validity of ICD-9-CM and ICD-10 administrative data in recording clinical conditions in a unique dually coded database." Health Serv Res **43**(4): 1424-1441.

Yang, H., et al. (2020). "Pre-pandemic psychiatric disorders and risk of COVID-19: a UK Biobank cohort analysis." Lancet Healthy Longev **1**(2): e69-e79.

Taquet, M., et al. (2021). "6-month neurological and psychiatric outcomes in 236 379 survivors of COVID-19: a retrospective cohort study using electronic health records." Lancet Psychiatry **8**(5): 416-427.

Kompaniyets, L., et al. (2021). "Body Mass Index and Risk for COVID-19-Related Hospitalization, Intensive Care Unit Admission, Invasive Mechanical Ventilation, and Death - United States, March-December 2020." MMWR Morb Mortal Wkly Rep **70**(10): 355-361.

Zhang, X., et al. (2021). "A systematic review and meta-analysis of obesity and COVID-19 outcomes." Sci Rep **11**(1): 7193.

Mahamat-Saleh, Y., et al. (2021). "Diabetes, hypertension, body mass index, smoking and COVID-19-related mortality: a systematic review and meta-analysis of observational studies." BMJ Open **11**(10): e052777.

Brockhattingen, K. K., et al. (2020). "Association between number of medications and mortality in geriatric inpatients: a Danish nationwide register-based cohort study." Eur Geriatr Med **11**(6): 1063-1071.

Watson, O. J., et al. (2022). "Global impact of the first year of COVID-19 vaccination: a mathematical modelling study." Lancet Infect Dis **22**(9): 1293-1302.

Curtis, H. J., et al. (2022). "Trends and clinical characteristics of COVID-19 vaccine recipients: a federated analysis of 57.9 million patients' primary care records in situ using OpenSAFELY." Br J Gen Pract **72**(714): e51-e62.

## Reviewer #4

The manuscript has great public health value and well presented. Just I have the following few comments.

**Thank you very much!**

1.	Why authors used categorical data instead of continuous scores?

**We are not sure what continuous scores the reviewer is referring to. We relied on discharge diagnosis data (except age and deprivation index) and therefore all covariates were defined with use of binary or categorical variables for age and deprivation index. Regarding the Charlson Comorbidity Index, we used each of its components as covariates because sample size is very large (compared to clinical studies), model fitting can only be better (multiple comorbidities vs. one index categorized in x classes), and the level of associations of different somatic comorbidities with COVID-19-related mortality is of interest per se (internal validity of the study results).**

2.	On page 7 and 8, the authors stated that "Our case study of salvage therapy suggests that triage decisions for life-saving measures at hospital were disproportionately taken to maximize health benefits at the expense of COVID-19 patients with mental disorders: salvage therapy rates due to caseload surges were significantly higher than expected in patients without mental disorders (+4.2% [95% CI, 3.8-4.5]) and lower in patients with mental disorders (-4.1% [95% CI, -4.4;-3.7]". What can be the possible explanations for this finding? can stigma against people with mental illness also be one possible explanation?

**We agree with the reviewer that our study results may also point to stigmatization and discrimination of mental health, though there might be other explanations such as less support from family, carers, and friends in medical decisions. We added these possible explanations In the revised manuscript (Discussion, page 10 lines 376-378 of the “marked-up” revision).**

3.	Title of the tables and graphs should give full information.

**We have provided more information in the Titles in the revised manuscript.**
